# Glycodendrimers and Modified ELISAs: Tools to Elucidate Multivalent Interactions of Galectins 1 and 3

**DOI:** 10.3390/molecules20047059

**Published:** 2015-04-20

**Authors:** Mark Wolfenden, Jonathan Cousin, Pratima Nangia-Makker, Avraham Raz, Mary Cloninger

**Affiliations:** 1 Department of Chemistry and Biochemistry, Montana State University, Bozeman, MT 59717, USA; E-Mails: mlwolfenden@gmail.com (M.W.); jon.cousin@chemistry.montana.edu (J.C.); 2 The Departments of Oncology and Pathology, School of Medicine, Wayne State University, Detroit, MI 48201, USA; E-Mails: makkerp@karmanos.org (P.N.-M.); raza@karmanos.org (A.R.)

**Keywords:** ELISA, galectin-1, galectin-3, glycodendrimer, dendrimer, multivalent

## Abstract

Multivalent protein-carbohydrate interactions that are mediated by sugar-binding proteins, *i.e.*, lectins, have been implicated in a myriad of intercellular recognition processes associated with tumor progression such as galectin-mediated cancer cellular migration/metastatic processes. Here, using a modified ELISA, we show that glycodendrimers bearing mixtures of galactosides, lactosides, and *N*-acetylgalactosaminosides, galectin-3 ligands, multivalently affect galectin-3 functions. We further demonstrate that lactose functionalized glycodendrimers multivalently bind a different member of the galectin family, *i.e.*, galectin-1. In a modified ELISA, galectin-3 recruitment by glycodendrimers was shown to directly depend on the ratio of low to high affinity ligands on the dendrimers, with lactose-functionalized dendrimers having the highest activity and also binding well to galectin-1. The results depicted here indicate that synthetic multivalent systems and upfront assay formats will improve the understanding of the multivalent function of galectins during multivalent protein carbohydrate recognition/interaction.

## 1. Introduction

Multivalent protein-carbohydrate interactions generally rely on multiple points of recognition/binding to enhance the individual binding interaction between one carbohydrate and its receptor, which are typically weak interactions [1]. A variety of synthetic multivalent scaffolds including linear polymers [2,3,4], star [5,6,7] and hyperbranched [8,9,10] polymers, gold nanoparticles [11,12,13], dendrimers [14], proteins [15], beads [16] and surfaces [17,18,19,20] have been functionalized with carbohydrates and then applied to the study and the mediation of multivalent protein-carbohydrate interactions [21]. These carbohydrate functionalized scaffolds have been used to study biological processes such as oncologic cellular aggregation [22] viral cell attachment [15,23], bacterial recognition [24], and signal transduction [25]. Applications for these glycosystems will no doubt become more widespread as the understanding of the roles of multivalent protein carbohydrate interactions in complex biological systems is improved [26]. 

One of the challenges of studying protein-carbohydrate interactions is the development of appropriate assays for assessment of heterogeneous binding interactions. Surface plasmon resonance (SPR) is very useful for studying binding interactions of lectins with surface-adhered glycans under flow [27,28]. Measurements are based on mass-change as lectins are bound and eluted from the glycan-adhered surface. Back scattering interferometry is a sensitive technique for studying lectin/glycan binding interactions both in solution and with surface-immobilized glycans, and detects the act of complexation without regard to size of the species [29]. Fluorescence binding assays, such as fluorescence resonance energy transfer (FRET) [30,31] or fluorescence lifetime (FL) [32,33], can be used to study thermodynamic binding interactions between disparately labelled lectin and glycan. Fluorescent binding assays can be expanded to a microarray format to analyze the influence of glycan valency, structure, and presentation on lectin binding [30,34]. Dynamic light scattering (DLS) is useful for studying the size of glycan/protein nanoparticles formed in solution [35,36,37,38]. This technique requires large amounts of sample and works best for relatively monodisperse nanoparticles. Fluorescence microscopy (FM), through fluorescent labelling of the protein or the glycoconjugate, can also be used to measure nanoparticle size [35]. Fluorescent labelling of cell-surface glycans can be used to investigate lectin-induced redistribution of glycans [39]. Isothermal titration calorimetry (ITC) is commonly used to measure binding constants in solutions [40,41]. ITC, however, requires large amounts of sample and is limited by the formation of insoluble protein/carbohydrate aggregates. A hemagglutination assay (HA) provides a model to study cellular adhesion mediated by lectin interactions with oligosaccharides expressed on the surface of red blood cells in a microtiter plate format, and can be used to detect the presence of a lectin or measure the minimum concentration of lectin to induce agglutination [42]. Precipitation assays are useful for studying the stoichiometry of lectin/glycan aggregates that precipitate out of solution but require large amounts of each species [2]. Enzyme linked immunosorbent assays (ELISAs) are relatively simple, high throughput platforms to detect the occurrence of binding interactions between lectins and surface-adhered glycans [43]. 

Clearly, due to the complexity of multivalent protein-carbohydrate interactions, a wide spectrum of assays has been developed. Some of the assays mimic a cell surface (SPR, BSI, ELISA), while others are intended to mimic non-surface bound multivalent assemblies of proteins with oligosaccharides (ITC, FL, DLS). Assays that incorporate cells (FM, HA) and assays that take advantage of phase changes upon binding (precipitation assay) have also been applied. All of these assays are valuable for elucidating the roles that multivalent protein-carbohydrate interactions have in biology and for determining the selectivity and specificity of interactions.

Here, we develop a straightforward assay for the evaluation of lectin(s) binding to/by members of a family of synthetic multivalent carbohydrates without the need to use specialized equipment. Systematic variations in the carbohydrate ligands and the size of structures should be easy to accommodate, enabling binding studies even for lectins that are not robust enough to be tethered to a surface or to be exposed to multiple washing/rejuvenation steps between trials. Taking these parameters into consideration, both SPR and ELISA would be good methods. Because we want to use a system in the absence of flow [44,45], we investigated the use of ELISAs for the systematic study of protein-carbohydrate interactions. Nuances associated with SPR, such as rebinding and model fitting, may provide a complementary but different analysis than the ELISA’s reported here.

**Figure 1:**
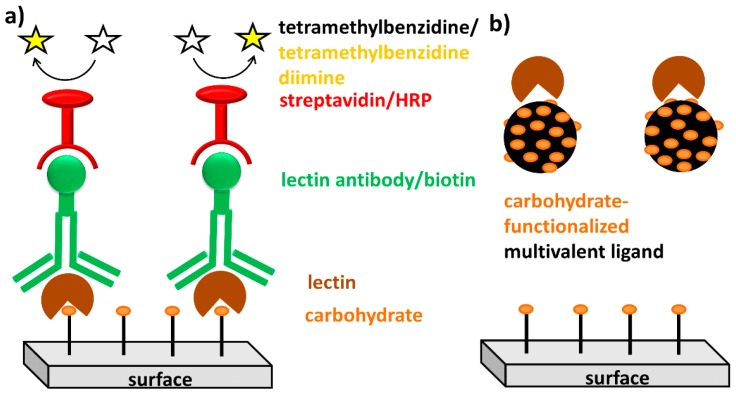
(**a**) Traditional ELISA with saccharide functionalized surface. Lectin binding is indicated by enzymatic oxidation of TMB, which produces a measurable color change; (**b**) Inhibition of lectin binding by a synthetic multivalent glycoconjugate is indicated by the absence of TMB oxidation.

The use of ELISAs to study protein-carbohydrate interactions has already been reported for many systems, making this assay a good choice for further development [43,46,47,48,49,50,51,52]. Generally, a solid surface is functionalized by a layer of carbohydrates [53]. The lectin of interest is bound to this surface, and binding is detected using an antibody and a detection system such as horseradish peroxidase [53,54]. After lectin binding to the carbohydrate functionalized surfaces has been established, the synthetic multivalent systems are introduced to the lectin [55]. Following washing, the synthetic multivalent system is identified as a lectin binder. However, if binding is only partially or is not inhibited, then the synthetic multivalent system is determined to be only weakly bound or not bound by the lectin. Representative ELISA binding and inhibition experiments are presented in Figure 1a,b. 

**Figure 2:**
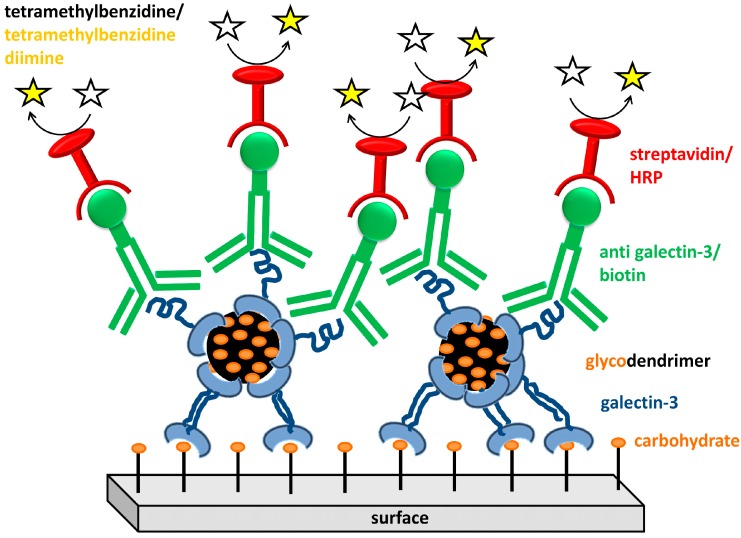
Multivalent complex formed with galectin-3 upon the addition of glycodendrimer. Using a traditional ELISA format with a carbohydrate-functionalized surface, glycodendrimers were observed to recruit galectin-3 rather than inhibit surface binding, which resulted in high absorbance measurements.

For many lectins, the existing ELISA protocols work very well. For galectins, however, the ELISA and inhibition ELISA protocols could not be applied to the study of multivalent binding. We found that as soon as the smallest possible amount of glycodendrimer was added to an inhibition ELISA with galectin-3, a multivalent complex was formed that actually enhanced binding of the tested galectin to the surface (Figure 2). 

Using surface-immobilized synthetic glycodendrimers, Andre *et al.* investigated the influence of glyconjugate valency on galectin-1 and galectin-3 binding affinity [56]. Homodimeric galectin-1 exhibited increased binding affinity with increased ligand valency, while monomeric galectin-3 afforded only modest affinity enhancements with increasing valency. Based on this important precedence, our goal was to develop an ELISA protocol that can be used to rapidly and effectively determine the relative binding affinity of galectins (and other lectins) for a range of synthetic multivalent frameworks on a surface. We adsorbed to the surface a series of glycodendrimers of different generations and bearing carbohydrates with a range of binding affinities for the galectins. The assay design, results from the assay, surface characterization using X-ray photoelectron spectroscopy, and the synthesis of the glycodendrimers that were required to systematically test the assay are reported here. 

## 2. Results and Discussion

Galectins-1 and -3 are members of the galectin family of galactoside-binding lectins [57,58,59]. Galectin-3 has a carbohydrate recognition domain and an N-terminal domain that is believed to enable aggregated states of the protein to occur [60]. Galectin-3 has been reported to be involved in mechanisms that cluster cell surface glycoproteins [61,62], cross-link receptors [63,64], and form lattices and larger aggregates [65], and these processes are involved in cellular function [66]. Galectin-1 is a prototype galectin that is found as a noncovalent dimer with monomeric subunits anchored such that the carbohydrate binding domains are located on opposing faces [57]. Similarly, galectin-1 has been reported to be involved in cell surface interactions that mediate cellular function [67,68,69,70,71]. Considering the complicated multivalent structures and the many multivalent functions of galectin-1 and galectin-3, it is not surprising that traditional ELISAs and inhibition ELISAs are insufficient for studying their multivalent binding to synthetic multivalent frameworks. 

### 2.1. Glycodendrimers

**Scheme 1:**
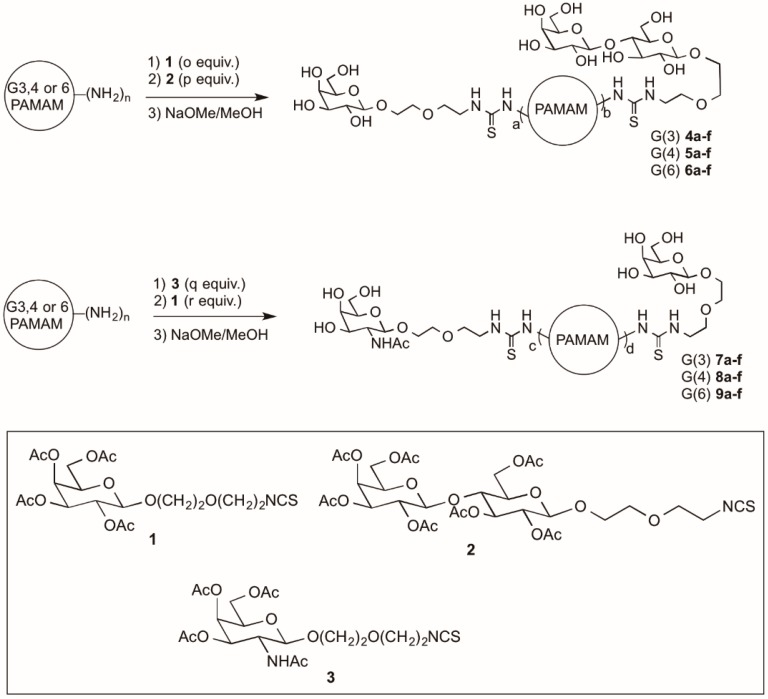
Synthesis of carbohydrate functionalized PAMAM dendrimers. (Values for **a**–**d** are shown in Table 1 and Table 2; numbers for o-r are given in the Experimental Section).

For the modified ELISAs, carbohydrate functionalized dendrimers **4**–**9**, which are shown in Scheme 1, were adsorbed to polystyrene surfaces. To obtain the requisite dendrimers, sequential addition of compounds **1**, **2**, and **3** was performed (Scheme 1). 

The synthesis of **2** has been previously reported [35], and the experimental methods for the syntheses of **1** and **3** are described in the Experimental Section. The degree of functionalization of the dendrimers with carbohydrates was determined by MALDI-TOF MS. The average amounts of sugars that were incorporated are shown in Table 1 and Table 2. 

The number of sugars attached to the dendrimer was determined using the change in the weight averaged molecular weight, M_w_, after addition of the carbohydrates and the change in M_w_ after deacetylation.

**Table 1:** Results for Compounds **4a**–**f**, **5a**–**f** and **6a**–**f** with galectin-3.

Compound Number	PAMAM Generation	Number of Galactose Sugars (a) ^a^	Number of Lactose Sugars (b) ^a^	Total Number of Sugars	IC_50_ (mM) Lactose	Maximum Absorbance Value
**4a**	3	0	24	24	0.31	1.03
**4b**	3	7	20	27	0.21	1.56
**4c**	3	14	12	26	0.12	1.33
**4d**	3	19	8	27	0.09	0.93
**4e**	3	24	3	27	0.12	0.66
**4f**	3	26	0	26	0.05	0.39
**5a**	4	0	57	57	0.15	1.28
**5b**	4	17	36	53	0.19	1.32
**5c**	4	28	25	53	0.20	1.02
**5d**	4	34	14	48	0.19	0.97
**5e**	4	44	7	51	0.28	0.84
**5f**	4	57	0	57	0.15	0.60
**6a**	6	0	130	130	0.09	0.84
**6b**	6	37	102	139	0.20	0.77
**6c**	6	59	83	142	0.14	0.75
**6d**	6	83	55	138	0.15	0.77
**6e**	6	105	38	142	0.12	0.73
**6f**	6	145	0	145	0.11	0.38

^a^ Numbers a and b correspond to the sugar loading shown in Scheme 1.

**Table 2:** Results for Compounds **7a**–**f**, **8a**–**f** and **9a**–**f** with galectin-3.

Compound Number	PAMAM Generation	Number of Galactose Sugars (c) ^a^	Number of GalNAc Sugars (d) ^a^	Total Number of Sugars	IC_50_ (mM) Lactose	Maximum Absorbance Value
**7a**	3	21	5	26	0.32	1.11
**7b**	3	15	10	25	0.34	1.14
**7c**	3	11	15	26	0.32	1.14
**7d**	3	8	19	27	0.38	1.36
**7e**	3	2	24	26	0.32	0.98
**7f**	3	0	28	28	0.29	0.92
**8a**	4	46	10	56	0.19	0.82
**8b**	4	33	22	55	0.15	0.89
**8c**	4	25	31	56	0.16	0.82
**8d**	4	12	40	52	0.06	0.71
**8e**	4	7	49	56	0.06	0.82
**8f**	4	0	54	54	0.07	0.53
**9a**	6	109	32	141	0.35	0.81
**9b**	6	85	54	139	0.27	1.40
**9c**	6	56	85	141	0.40	1.25
**9d**	6	43	105	148	0.29	1.17
**9e**	6	9	134	143	0.29	1.07
**9f**	6	0	154	154	0.36	0.94

^a^ Numbers c and d correspond to the sugar loading shown in Scheme 1.

### 2.2. Modified ELISA Using Surface Adsorbed Glycodendrimers and Galectin-3

**Figure 3:**
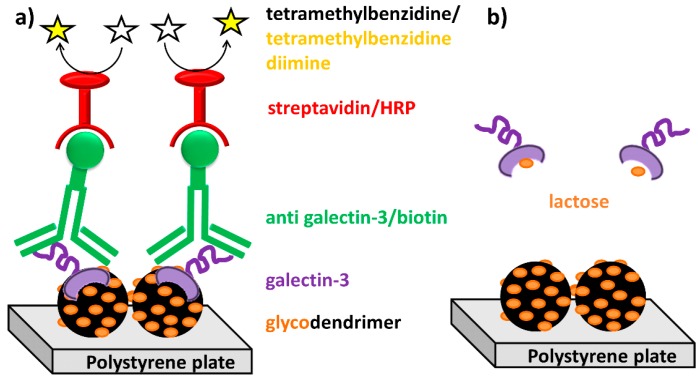
Modified ELISA to study galectin-3 interactions. (**a**) Glycodendrimers were adsorbed to the surface of a plate, and galectin-3 was added to measure binding affinity; (**b**) Monomeric lactose was added to inhibit galectin-3 binding to the glycodendronized surface.

The ELISA to study the dendrimer:galectin-3 binding interaction is based upon the dendrimer adsorbing to the 96 well plate and subsequently inhibiting the dendrimer:galectin-3 interaction with varying concentrations of lactose as shown in Figure 3. This method is similar to that reported by Roy, Gabius, and co-workers [56] and allowed for comparison of the relative binding associations of compounds **4a**–**f**, **5a**–**f**, **6a**–**f**, **7a**–**f**, **8a**–**f** and **9a**–**f** with galectin-3. Other ELISA methods were investigated such as adsorbing laminin and asialofetuin to the plate and then using the sugar functionalized dendrimers to inhibit the glycoprotein: galectin-3 surface interaction. However, these protocols inevitably resulted in very high galectin-3 binding to the surface. This result is suggestive of a cooperative glycodendrimer/galectin-3 system that caused galectin-3 to bind strongly to the plate even at very high dendrimer concentrations (Figure 2).

To measure the efficacy of the monomer carbohydrates for binding to galectin-3, an ELISA was performed with **4a**, **5a** and **6a** adsorbed to the 96 well plates, and galactose, GalNAc, lactose, and mannose were used separately as inhibitors of galectin-3 binding by the adsorbed glycodendrimers. Binding curves are shown in Figure S1 of the Supplementary Material. Previous studies have indicated different binding constants for various carbohydrates, both natural and chemically modified, with galectin-3 [72,73]. According to the ELISA inhibition assay using **4a** with various inhibitors and setting the relative IC_50_ value for lactose to 1, GalNAc and galactose had relative IC_50_ values of 111 and 90, respectively. Using **5a** with various inhibitors and setting the relative IC_50_ value for lactose to 1, GalNAc had a relative inhibitory potency value of 41 and galactose of 46. Using **6a** with various inhibitors and setting the relative IC_50_ value for lactose to 1, GalNAc had a relative inhibitory potency value of 40 and galactose of 50. This is in agreement with a report by Brewer *et al.* of a 66-fold increase in affinity for lactose over galactose using a hemagglutination inhibition assay [72]. Interestingly, there appeared to be little or no difference between the relative IC_50_ values for galactose and GalNAc. This suggests the *N*-acetyl group on GalNAc does not have a significantly different binding interaction than the 2'OH group on galactose upon binding to galectin-3. This is not entirely surprising considering the 2'OH position on lactose in X-ray crystallography studies appears to have no major interaction with galectin-3 [58]. However, hydrophobic substituents at the 2'OH position have afforded significantly increased binding constants relative to galactose for galectin-3 binding [74].

**Figure 4:**
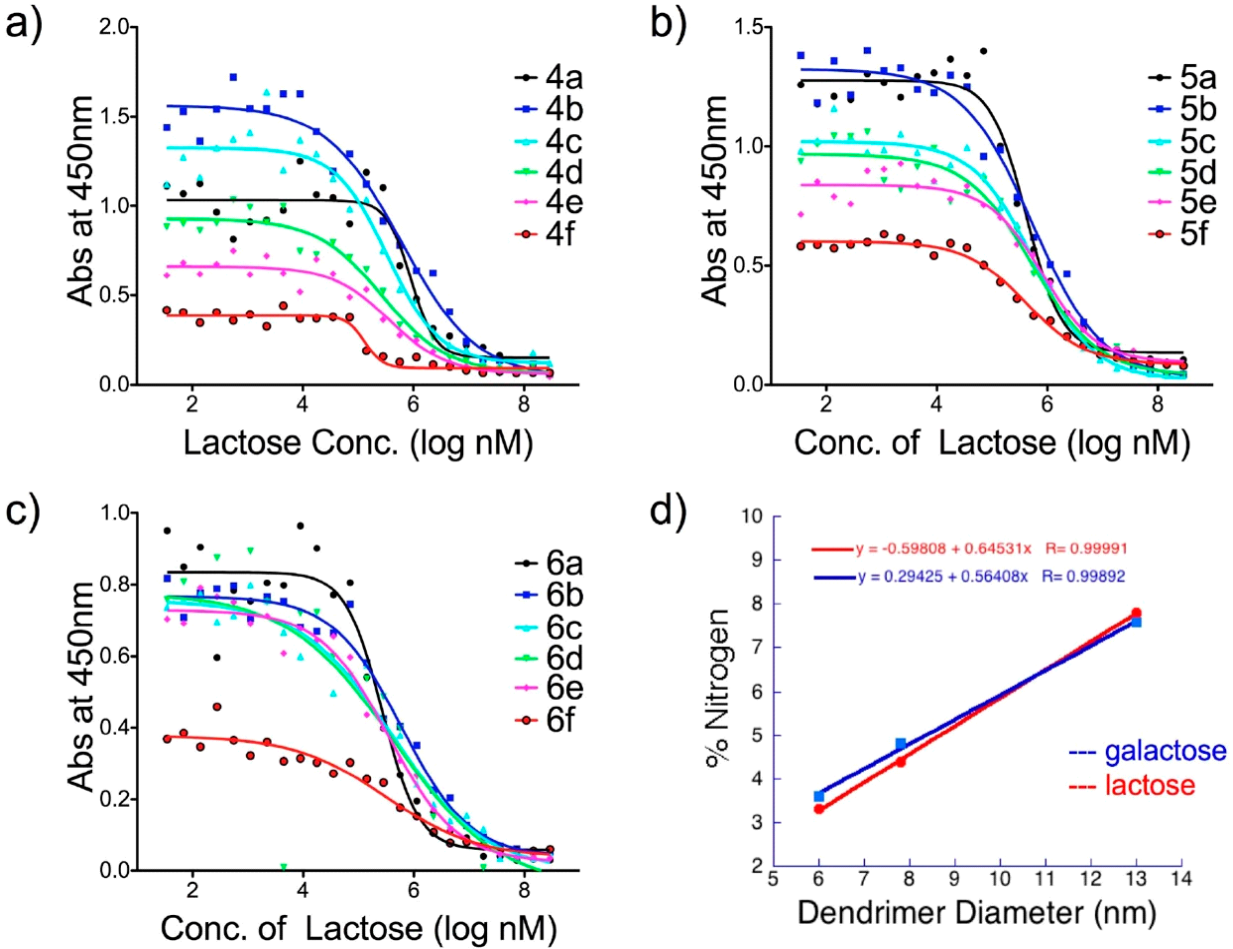
ELISA binding curves with (**a**) **4a**–**f**; (**b**) **5a**–**f**; (**c**) **6a**–**f** and (**d**) XPS results of dendrimer diameter *vs*. nitrogen % for compounds **4a**, **5a**, **6a** and **4f**, **5f**, **6f**.

ELISA binding curves and surface characterization information for galactose/lactose functionalized dendrimers are shown in Figure 4. The results of compounds **4a**–**f** binding with galectin-3 are shown in Figure 4a, and the IC_50_ lactose inhibition values are reported in Table 1. The IC_50_ values ranged from 0.05 mM for **4f** to 0.31 mM for **4a**. Shown in Figure 4b are the ELISA binding curves for **5a**–**f**, and the reported lactose inhibition values are reported in Table 1. These IC_50_ values ranged from 0.15 mM for **5a** and **5f** to 0.28 mM for **5e**. Shown in Figure 4c are the ELISA binding curves for **6a**–**f**, and the reported lactose IC_50_ values are provided in Table 1. These IC_50_ values ranged from 0.09 mM for **6a** to 0.20 mM for **6b**. Shown in Figure 4d is a comparison of surface adsorptions for dendrimers with different endgroups. Additional details about Figure 4d are provided in Section 2.4 below.

**Figure 5:**
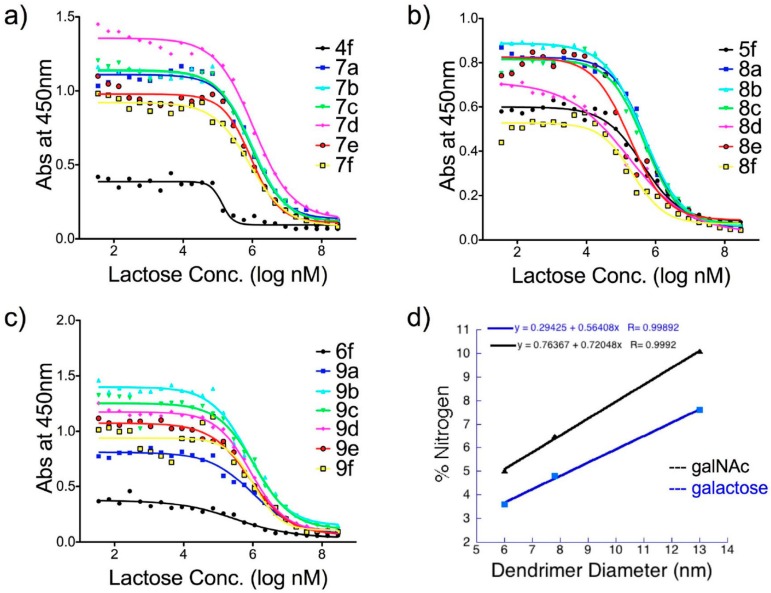
ELISA binding curves with (**a**) **4f**, **7a**–**f**; (**b**) **5f**, **8a**–**f**; (**c**) **6f**, **9a**–**f** and (**d**) XPS results of dendrimer diameter *vs*. nitrogen % for compounds **4f**, **5f**, **6f** and **7f**, **8f**, **9f**.

ELISA binding curves and surface characterization information for galactose/GalNAc functionalized dendrimers are shown in Figure 5. The ELISA binding curves of compounds **7a**–**f** are depicted in Figure 5a (lactose inhibiting concentrations are shown in Table 2). All of the measured lactose inhibiting concentrations were very similar, ranging from 0.29 mM (**7f**) to 0.38 mM (**7d**). For compounds **8a**–**f** the ELISA binding curves are shown in Figure 5b, and the lactose inhibiting concentrations are reported in Table 2. The IC_50_ values ranged from 0.06 mM for **8d** and **8e** to 0.19 mM for **8a**. Shown in Figure 5c are the ELISA binding curves for compounds **9a**–**f** and the reported lactose inhibiting concentrations are reported in Table 2. The IC_50_ values ranged from 0.27 mM for **9b** to 0.40 mM for **9c**. Shown in Figure 5d is a comparison of surface adsorptions for dendrimers with different endgroups. Additional details about Figure 5d are provided in Section 2.4 below.

These results suggest that the carbohydrates which were presented on the dendrimers **4**–**9** **a**–**f** did not have a great effect upon the binding affinity of the glycodendrimers toward galectin-3 as measured in a competitive ELISA based assay. In contrast, the amount of galectin-3 that was recruited by the surface bound dendrimer increased significantly with higher lactose loading. For example in Figure 2b, the maximum absorbance for **5b** is 1.32 and for **5f** is 0.60, and other compounds in this series follow an increasing trend in higher maximum absorbance values with higher lactose loadings. This trend is also apparent in the third generation series of lactose: galactose functionalized dendrimers (Figure 2a), with the exception of **4a**. The maximum absorbance value for **4b** was 1.56 and was 0.39 for **4f**, again with an increasing trend in maximum absorbance value with higher lactose loading. With sixth generation dendrimers, it appears that even with lower lactose loading, the galectin-3 recruitment does not change significantly. However, with fully galactose functionalized G(6), **6f**, the galectin-3 maximum absorbance is 0.38, which is much lower than the maximum absorbance values of the other G(6) lactose: galactose functionalized dendrimers (absorbance values for **6a**–**e** are 0.84, 0.77, 0.75, 0.77 and 0.73, respectively).

**Figure 6:**
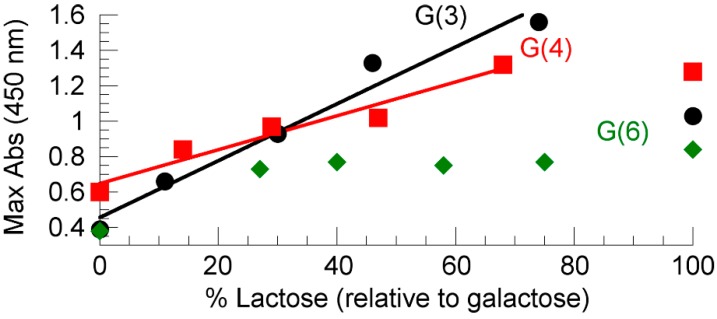
Percent lactose of the lactose/galactose functionalized dendrimers *vs.* maximum absorbance for binding of galectin-3 in a modified ELISA.

Previously, we showed that the activity of dendrimers toward concanavalin A, a model plant lectin that is commonly used to study multivalent protein-carbohydrate interactions, was linearly dependent on the ratio of low to high affinity ligands that were present on dendrimers [75]. In Figure 6, the linear correlation between the recruitment of galectin-3 to the dendronized surface and the ratio of lactose to galactose is clearly visible for third generation compounds **4b**–**f** and for fourth generation compounds **5b**–**f**. For the sixth generation series of dendrimers **6a**–**f**, the plot levels off upon functionalization of about 30% of the dendrimer endgroups with lactose. The difference in galectin-3 recruitment is negligible for addition of a further ninety lactosides (maximum absorbance values at 450 nm are very similar for **6a**–**e**), indicating that dendrimers can ultimately be functionalized with additional groups as needed without reducing the galectin-3/glycodendrimer binding interaction.

### 2.3. ELISA Using Surface Adsorbed Glycodendrimers with Galectin-1

**Figure 7:**
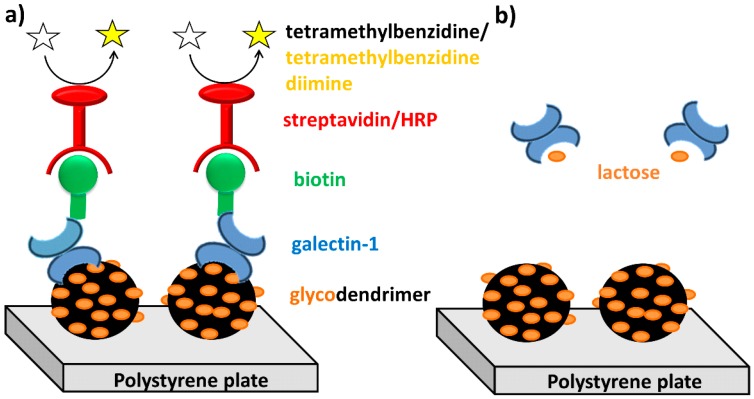
Modified ELISA to study galectin-1 binding interactions. (**a**) Glycodendrimers were adsorbed to the surface of a plate, and biotinylated galectin-1 was added to measure binding affinity; (**b**) Monomeric lactose was added to inhibit galectin-1 binding to the glycodendronized surface.

After using the modified ELISA to study galectin-3 binding interactions with glycodendrimers, we validated the broader utility of this assay by studying the binding of galectin-1 to lactose functionalized dendrimers adsorbed to the polystyrene surface. The design of the ELISA is basically the same as was used for galectin-3, except galectin-1 was biotinylated and the anti-galectin antibody was eliminated (Figure 7).

**Figure 8:**
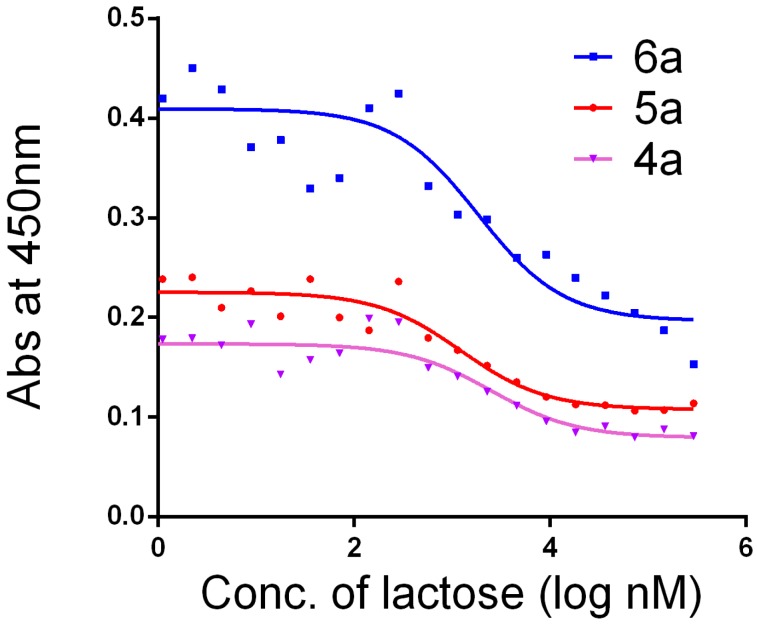
ELISA binding curve with **4a**, **5a**, and **6a**.

Galectin-1 binding to **4a**, **5a**, and **6a** was observed as shown in Figure 8. In addition to showing that galectin-1 does bind well to the glycodendrimers, relative differences among glycodendrimer architectures were evaluated by comparing the maximum absorbance values and the IC_50_ values for the three generations of glycodendrimers. Maximum absorbance values correspond to the relative amount of galectin-1 recruited by the glycodendrimers. The results indicate that larger glycodendrimers, which bear more ligands, recruit more galectin-1: the amount of galectin-1 recruited by **4** was less than half the amount recruited by **6**.

IC_50_ values calculated from the inhibition ELISA binding curves are shown in Table 3. The highest IC_50_ value with galectin-1 was for third generation lactose functionalized dendrimer **4a**. IC_50_ values for fourth and sixth generation glycodendrimers **5a** and **6a** were about 30% lower, and the range in IC_50_ values was narrow, indicating that a consistent binding interaction occurs for all generations of glycodendrimers.

**Table 3:** Results for Compounds **4a**, **5a**, and **6a** with galectin-1.

Compound Number	PAMAM Generation	Number of Lactose Sugars	IC_50_ (nM) Lactose	Maximum Absorbance Values
**4a**	3	20	2.52	0.17
**5a**	4	45	1.85	0.23
**6a**	6	100	1.74	0.41

### 2.4. Characterization of Surfaces after Adsorption of Glycodendrimers

X-ray photoelectron spectroscopy (XPS) was used to analyze the amount of the glycodendrimer that adsorbs to the polystyrene plates that were used in the ELISAs. Figure 4d shows the amount of nitrogen, which was used to analyze dendrimer coating, in comparison to the dendrimer diameters (as reported previously by this group [76]) for lactose and galactose functionalized dendrimers. The linear relationship between dendrimer radius and nitrogen content indicates that the same amount of dendrimer is adsorbed to the surface regardless of which carbohydrate (lactose or galactose) was present. As shown in Figure 4d and Figure 5d, the linear relationship between dendrimer radius and nitrogen content is consistent, with R values of 0.9999 for **4a**, **5a** and **6a**, 0.9989 for **4f**, **5f** and **6f**, and 0.9992 for **7f**, **8f** and **9f**. Compounds **4a**–**6a** and **4f**–**6f** have the same amount of nitrogen and gave the same amounts of nitrogen signal relative to the dendrimer diameter. Compounds **7f**–**9f** have the 2-NHAc group, and this increases the nitrogen signal. Within the fourth generation dendrimer series, **5f** and **5a** would theoretically contain 303 nitrogens and **8f** would contain 358 nitrogens (assuming perfect PAMAM dendrimers). These values correspond to an 18% increase in nitrogen signal for GalNAc over lactose or galactose functionalized dendrimers. However, in the XPS experiments, the increase in the nitrogen signal for **8f** was significantly higher than 18%. This was also observed with the G(3) and G(6) series. As shown in Figure 5d, the trend for the GalNAc functionalized dendrimers is also consistent with dendrimer diameter, but dendrimer adsorption is consistently higher for GalNAc functionalized dendrimers than it is for the galactose and lactose functionalized dendrimers. The amount of galactose functionalized dendrimers **7**–**9a** and lactose functionalized dendrimers **4**–**6a** adsorbed to the surface was shown by XPS to be equal and consistent, indicating formation of a monolayer. The GalNAc functionalized dendrimers **7**–**9f** appeared to adsorb somewhat better, but monolayer formation, as shown by the linearity of the relationship between the radius of the dendrimer and the concentration of nitrogen, is still indicated. This increase in adsorption by GalNAc functionalized dendrimers is in agreement with the higher maximum absorbance values that were observed in the modified ELISAs for these compounds. The XPS experiments suggest that the higher galectin-3 recruitment that was observed for glycodendrimers that had higher GalNAc loadings when compared to dendrimers with more galactose residues was due to differences in adsorption rather than differences in protein binding affinity.

Computer simulations suggest that dendrimers lie flat on a surface and that increased interaction strength results in spreading out and flattening of the dendrimers [77]. These simulations were supported by subsequent studies with transmission electron microscopy, which showed that surface adsorbed dendrimers adopt a circular shape [78]. When glycosides are conjugated to the PAMAM backbone, the resulting glycodendrimers adopt a circular shape with a slight declivity in the center upon surface adsorption [76]. Ligands are likely located in a volume shell on the periphery of the donut-shaped, surface adsorbed glycodendrimers, and the lectins cluster around the exposed surface [79].

### 2.5. Discussion

Although the preferred aggregation state of galectin-3 at the cell surface remains obscure, the modified ELISA results displayed here show very similar binding avidity for all the dendrimers, which indicates that the interaction between galectin-3 and glycodendrimers is not monomeric. If the glyco-dendrimer/galectin-3 interaction were monomeric, then the results with glycodendrimers would resemble the results with the monomer inhibitors, and they do not. The trends of increasing galectin-3 recruitment with higher lactose loading suggest that another (not monomeric) process is playing a large role in how these surface bound dendrimers interact with galectin-3. The capacity of the dendrimer to bind galectin-3 was observed to increase when the dendrimers were functionalized with higher affinity carbohydrates compared to results with glycodendrimers bearing lower affinity carbohydrates. This process of multivalently recruiting varying amounts of galectin-3 by the glycodendrimers could be similar to a cell surface receptor cluster mechanism. The ELISA results indicate that lectin recruitment may have a powerful role in clustering specific receptors.

Two properties of the galectin-1 binding interaction with glycodendrimers are evident from the binding assays. First, galectin-1 recruitment was valency-dependent in a manner similar to galectin-3, with more galectin-1 recruitment occurring for higher generation dendrimers. Second, inhibition of the galectin-1/glycodendrimer interaction by monomeric lactose was similar regardless of the size/generation of the glycodendrimer. The similarity in the IC_50_ values observed for **4a**, **5a**, and **6a** suggests that galectin-1 was bound to each generation with a similar affinity regardless of the fundamental architectural differences in size, valency, and ligand density among the dendrimer generations.

We recognize that the modified ELISA performed here might be somewhat artificial due to the washing steps and tower building inherent in an ELISA. However the similarity in IC_50_ values for galectin-3 and surface bound dendrimer with very different sugar epitopes, as well as the similarity in IC_50_ values for galectin-1 binding to different generations of lactose functionalized dendrimers, require an explanation that goes beyond monomeric interaction. This explanation could be similar to the “bind and slide” mechanism presented by Brewer and co-workers, which could explain the trend in maximum galectin recruitment [80]. An equilibrium in which galectin-3 binds preferentially to the higher affinity sugars on the heterogeneously functionalized glycodendrimers is also likely, and as evidenced by the higher recruitment of galectin-3 when there are higher affinity lactose sugars available. The modified ELISA results reported here indicate that a multivalent mechanism is at play [1], most likely involving an interplay of different effects such as lectin clustering [76,81,82], bivalent (or higher order) interactions [34,83] and statistical concentration effects [84,85].

## 3. Experimental Section

### 3.1. General Methods

General reagents were purchased from Acros Organics (Pittsburgh, PA, USA) and Aldrich Chemical Company (Milwaukee, WI, USA). PAMAM dendrimers were purchased from Dendritech (Midland, MI, USA). Methylene chloride was purified on basic alumina; other solvents were used as received. 32–63 μ “40 micron flash” silica gel for flash column chromatography purification was purchased from Scientific Adsorbents Incorporated (Atlanta, GA, USA).

### 3.2. Matrix Assisted Laser Desorption Ionization Time of Flight MS (MALDI-TOF)

MALDI mass spectra were acquired using a Biflex-III time-of-flight mass spectrometer (Bruker, Billerica, MA, USA). Spectra of all functionalized dendrimers were obtained using a *trans*-3-indoleacrylic acid matrix with a matrix-analyte ratio of 3000:1 or 1000:1. Bovine serum albumin (molecular weight, MW, 66,431 g/mol), cytochrome C (MW 12,361 g/mol), and trypsinogen (MW 23,982 g/mol) were used as external standards. An aliquot corresponding to 12–15 pmol of the analyte was deposited on the laser target. Positive ion mass spectra were acquired in linear mode and the ions were generated by using a nitrogen laser (337 nm) pulsed at 3 Hz with a pulse width of 3 nanoseconds. Ions were accelerated at 19–20,000 volts and amplified using a discrete dynode multiplier. Spectra (100 to 200) were summed into a LeCroy LSA1000 high-speed signal digitizer. All data processing was performed using the Bruker XMass/XTOF V 5.0.2 software (Bruker, Billerica, MA, USA). Molecular mass data and polydispersities (PDI) of the broad peaks were calculated by using the Polymer Module included in the software package. The peaks were analyzed using the *continuous* mode. MALDI-TOF MS spectra were obtained after each addition of isothiocyanate, and the change in M_w_ upon the first addition was divided by the M_w_ of the isothiocyanato carbohydrate (galNAc 476 g/mol, galactose 477 g/mol) to give a quantity that is denoted here as A (Equation (1)). The total number of carbohydrate residues added for the second addition (B in Equation (2)) was determined by subtracting M_w_ for unfunctionalized PAMAM from the M_w_ after each sequential additions of isothiocyanate and then dividing by the M_w_ of that carbohydrate (galactose—477 g/mol, galNAc—476 g/mol, lactose 765 g/mol), and this sum was the total, shown as C (Equation (3)). The total number of carbohydrate residues was also determined by dividing the change in M_w_ upon deacylation by 168 (the loss of four acetyl groups per galactose), 126 (the loss of three acetyl groups per galNAc) or 294 (the loss of seven acetyl groups per lactose) and this number is denoted as D (Equation (4)). The total number of carbohydrate residues was determined again by dividing the change in M_w_ upon deacylation from the PAMAM dendrimer, by the M_w_ of the deacetylated tethered sugar (309 for galactose, 352 for galNAc and 471 for lactose), denoted by as E (Equation (5)). These three methods of determining the total sugar loading were then averaged, denoted as F (Equation (6)). The number of residues of the first sugar was the corrected by dividing A by C times F to give A' as shown in Equation (7), to obtain the most accurate value for how many residues of the first isothiocyanato sugar (and also by difference for how many residues of the second isothiocyanato sugar) were added to the dendrimer. Sample numbers using data from compound **4d** are provided in the equations below:

(1) $$A=\;\frac{\mathrm{Mw}\;\left(\mathrm{all}\;1\;\mathrm{addition}\right)\;-\;\mathrm{Mw}\;\left(\mathrm{PAMAM}\right)}{477}=\;\frac{15800-6800}{477}=18.9$$

(2) $$B=\;\frac{\mathrm{Mw}\;\left(\mathrm{all}\;2\;\mathrm{additions}\right)\;-\;\mathrm{Mw}\;\left(\mathrm{PAMAM}\right)}{765}=\frac{21800-15800}{765}=7.8$$

(3) C=total # sugars=A+B=18.9+7.8=26.7

(4) $$D=\;\frac{\mathrm{Mw}\;\left(\mathrm{all}\;\mathrm{RNCS}\;\mathrm{added}\right)\;-\;\mathrm{Mw}\;\left(\mathrm{deacetylated}\right)}{\;\;\;\;\;\;\;\;\;\;\;\;\;\;\;\;\;\frac{\left[\left(A\;\times \;168\right)\;+\;\left(B\;\times \;294\right)\right]}{C}}=\;\frac{21800-15700}{\left[\frac{5469}{26.7}\right]}=29.8$$

(5) $$E=\;\frac{\mathrm{Mw}\;\left(\mathrm{deacetylated}\right)\;-\;\mathrm{Mw}\;\left(\mathrm{PAMAM}\right)}{\left[\frac{\left(A\;\times \;309\right)+\left(B\;\times \;471\right)}{C}\right]}=\;\frac{15700-6800}{\left[\frac{9514}{26.7}\right]}=25.0$$

(6) $$F=\;\frac{\left(C+D+E\right)}{3}=27.1$$

(7) $$A^{\prime }=\left(A/C\right)\;\times \;F=\;\left(18.9/26.7\right)\;\times \;27.1=19.2$$

(8) $$B^{\prime }=\;\left(B/C\right)\;\times \;F=\;\left(7.8/26.7\right)\;\times \;27.1=7.9$$

### 3.3. NMR

^1^H-NMR spectra were recorded on DPX 300 (300 MHz) and DPX-500 (500 MHz) spectrometers (Bruker). Chemical shifts are reported in ppm from tetramethylsilane with the residual protic solvent resonance as the internal standard (chloroform: δ 7.25 ppm; dimethyl sulfoxide: δ 2.50 ppm). Data are reported as follows: chemical shift, multiplicity (s = singlet, bs = broad singlet, d = doublet, t = triplet, q = quartet, p = pentet, m = multiplet, app = apparent), integration, coupling constants (in Hz) and assignments. ^13^C-NMR spectra were recorded on a Bruker DPX 500 (125 MHz) spectrometer with complete proton decoupling. Chemical shifts are reported in ppm from tetramethylsilane with the solvent as the internal standard (CDCl_3_: δ 77.0 ppm).

### 3.4. Biotinylation of Galectin-1

The biotinylated procedure was adopted and modified from the Thermo Scientific Instructions, EZ-Link^®^ Sulfo-NHS-LC-Biotin. 21327, 1855.4 (2011). Biotinylation of galectin-1 was achieved with EZ-Link^®^ Sulfo-NHS-LC-Biotin (sulfosuccinimidyl-6-[biotin-amido]hexanoate) (Thermo Scientific^®^, Waltham, MA, USA). A stock solution of Sulfo-NHS-LC-Biotin was prepared at 1 mg/mL in PBS (pH 7.4, 15 mM NaCl). To a solution of galectin-1 in PBS, the biotin reagent was added in an 8 molar excess, and the reaction was stirred at RT for 3 h. After 3 h, the reaction mixture was purified by dialysis against PBS (pH 7.4, 15 mM NaCl) with a 1 kD MWCO (Spectrum Laboratories, Inc., Houston, TX, USA, 6 Spectra/Por^®^ Dialysis Membrane).

Quantitation of biotin loading was achieved with a Biotin Quantitation Kit (Pierce^®^, Life Technologies, Thermo Scientific, Waltham, MA, USA) and spectrometric measurements were made using a SoftMax^®^ Pro 5 SPECTRA max Plus 3.84 (Molecular Devices, Sunyvale, CA, USA, Serial No.: SMP500-14501-EARG). To a 1 mL cuvette (PLASTIBRAND^®^, Thermo Scientific, Waltham, MA, USA), 800 µL of PBS buffer (pH 7.4, 15 mM NaCl) was added and the spectrophotometer was zeroed at 500 nm. To a microtube of 4'-hydroxyazobenzene-2-carboxylic acid (HABA)/Avidin Premix, equilibrated to room temperature (66.7 °C), 100 µL of millipore H_2_O was added and mixed with a pipette until solubilization. The HABA/Avidin Premix solution was added to the 1 mL cuvette containing the PBS buffer and mixed. The absorbance of the HABA/Avidin solution was measured at 500 nm (A_500_ H/A). To the cuvette, 100 µL of biotinylated galectin-1 was then added and mixed thoroughly. The absorbance of the HABA/Avidin/biotinylated galectin-1 mixture was measured at 500 nm (A_500_ H/A/B-Gal-1. From the change in absorbance at 500 nm, the number of moles of biotin per mole of galectin-1 was calculated using Beer’s Law, to yield 5 moles biotin per galectin-1.

### 3.5. Synthesis Protocols

#### 3.5.1. Synthesis of Compounds **1** and **3**

**Scheme 2:**
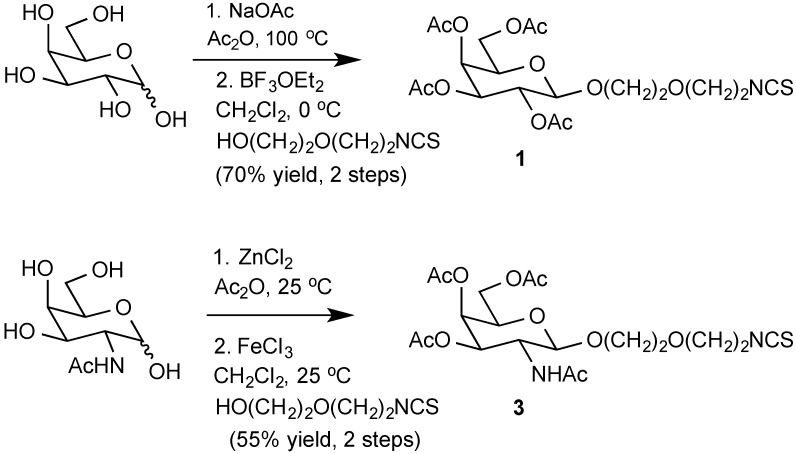
Synthesis of isothiocyanato-functionalized galactose **1** and galNAc **3**.

Compounds **1** and **3** were synthesized as shown in Scheme 2.

*1-O-(5-Isothiocyanato-3-oxopentyl)-2,3,4,6-tetra-O-acetyl-β-d-galactopyranoside* (**1**). 1,2,3,4,6-Penta-*O*-acetyl-β-d-galactopyranoside (2.0 g, 5.1 mmol) was dissolved in methylene chloride (50 mL) with 2-(2-isothiocyantoethoxy)ethanol (1.5 g, 10.2 mmol) and cooled to 0 °C followed by addition of BF_3_OEt_2_ (1.4 g, 10.2 mmol) via syringe pump over 30 min. The reaction was stirred for 1 h, at which point NaHCO_3_ (1 g) was added. The mixture was filtered over Celite and solvent was removed *in vacuo*. The residue was purified via column chromatography with a 1:1 ethyl acetate–hexane eluent (R_f_ 0.25), yielding 1.8 g (76% yield) of pure material. ^1^H-NMR (300 MHz, CDCl_3_) δ 5.38 (1H, d, *J* = 2.1 Hz, H4), 5.19 (1H, app t, *J* = 7.8 Hz, H2), 5.02 (1H, dd, *J* = 2.1, 10.1 Hz, H3), 4.55 (1H, d, *J* = 7.8 Hz, H1), 4.11 (2H, m, H5), 3.95 (2H, m, H6), 3.68 (7H, m), 2.19 (3H, s), 2.05 (3H, s), 2.03 (3H, s), 1.96 (3H, s) ppm. As reported [86].

*1-O-(5-Isothiocyanato-3-oxopentyl)-2,3,4,6-tetra-O-acetyl-β-d-galactosaminopyranoside* (**3**). 0.94 g of 1,3,4,6-penta-*O*-acetyl-β-d-*N*-acetyl-galactaminopyranoside (2.4 mmol) was dissolved in 15 mL of methylene chloride, 1.4 g of dririte was added, the slurry stirred for 10 min and 1.4 g of FeCl_3_ (10.2 mmol) and 0.83 g of 2-(2-isothiocyantoethoxy)ethanol (5.7 mmol) were added. The reaction was stirred 24 h, then 1 g of NaHCO_3_ was added. The mixture was filtered over celite and solvent was removed in vacuo. The residue was purified via column chromatography with a 9:1 ethyl acetate–hexane eluant (R_f_ 0.4), yielding 0.70 grams of pure material. ^1^H-NMR (500 MHz DMSO*-d*_6_) δ 7.83 (1H, d, *J* = 9.2 Hz, N*H*Ac), 5.21 (1H, d, *J* = 3.0 Hz, H4), 4.97 (1H, dd, *J* = 3.0, 11.2 Hz, H3), 4.55 (1H, d, *J* = 9.5 Hz, H1), 4.02 (3H, m, H5, OC*H*_2_CH_2_O), 3.87 (1H, app q, *J* = 9.2 (N*H*Ac), 9.5, 11.2 Hz, H2), 3.80 (2H, t, *J* = 4.9 Hz, OCH_2_C*H*_2_NCS), 3.59 (6H, m, H6, CH_2_C*H*_2_OC*H*_2_CH_2_), 2.11 (3H, s), 2.04 (3H, s), 1.89 (3H, s), 1.78 (3H, s) ppm. As reported [87]. 

#### 3.5.2. Representative Procedure for the Synthesis of Acetyl Protected Lactose and Galactose Functionalized Dendrimers

An aqueous solution of amine terminated G(4)-PAMAM dendrimer (2.478 g of a 17% *w*/*w* solution in water, 421.2 mg, 31.2 μmol) was lyophilized to leave a foamy residue. DMSO (7.02 mL) was added to this residue to give a 60 mg/mL solution. A 300 mM solution of **1** (0.31 mL, 73 μmol, 35 mg) was added to the 60 mg/mL G(4) PAMAM dendrimer solution (0.5 mL, 30 mg, 4.40 μmol). The reaction was stirred for 8 h at which point a 75 μL aliquot was removed for MALDI-TOF analysis. After MALDI-TOF analysis indicated reaction completion, a 300 mM solution of **2** (0.35 mL, 85 μmol, 40.2 mg) in DMSO was added. The solution was then stirred for 8 h, lyophilized, and directly subjected to deacetylation conditions. The amounts of reagents used for individual compounds are listed in Table 4.

**Table 4:** Amounts used in synthesis of peracetylated pre-compounds **4a**–**6f**.

Compound Number	PAMAM Generation	PAMAM (μmol)	Galactose (μmol) (o ^a^)	Lactose (μmol) (p ^a^)
**4a**	3	4.4	0	140.8
**4b**	3	4.4	28.2	112.6
**4c**	3	4.4	56.3	84.5
**4d**	3	4.4	84.5	56.3
**4e**	3	4.4	112.6	28.2
**4f**	3	4.4	140.8	0
**5a**	4	2.2	0	140.8
**5b**	4	2.2	28.2	112.6
**5c**	4	2.2	56.3	84.5
**5d**	4	2.2	84.5	56.3
**5e**	4	2.2	112.6	28.2
**5f**	4	2.2	140.8	0
**6a**	6	0.5	0	120.0
**6b**	6	0.5	24.0	96.0
**6c**	6	0.5	48.0	72.0
**6d**	6	0.5	72.0	48.0
**6e**	6	0.5	96.0	24.0
**6f**	6	0.5	120.0	0

^a^ Values for o and p (as shown in Scheme 1 of the primary manuscript) derive directly from the μmol values shown here.

#### 3.5.3. General Deacetylation Procedure to Afford Dendrimers **4**–**6**

To the lyophilized solid peracetylated dendrimers, 1:1 water–methanol (1 mL) was added, at which point the dendrimer became a white precipitate. To this mixture was added NaOMe (0.2 equivalents, 0.8 M in MeOH) for each peripheral carbohydrate, and it was allowed to stir for 3 h. If, at this time, the mixture had not become a clear solution, a further 0.2 equivalents of NaOMe (0.8 M in MeOH) was added, and this step was repeated until the mixture became a clear and colorless solution. Aqueous HCl solution (0.1 M) was then added slowly until the pH was ~7. This neutralized solution was placed in a dialysis membrane (MW cutoff 3,500 Da) and dialyzed in 1 L of DI water for 8 h. The water was changed and let stand for a further 8 h twice more. The remaining liquid in the membrane was frozen and lyophilized to give a white fluffy solid. Compounds were characterized with ^1^H-NMR and MALDI-TOF MS. MALDI-TOF MS data is summarized in Table 5.

#### 3.5.4. Spectroscopic Data for Compounds **4**–**6**

**4a**: ^1^H-NMR (500 MHz *d*_6_-DMSO) δ 7.97 (1H, bs), 7.78 (0.9H, bs), 7.47 (1.5H, bs), 5.17 (1.3H, s), 5.11 (2.4H, m), 4.79 (1.5H, app t, *J* = 9.0 Hz), 4.69 (2.2H, m), 4.62 (1.1H, *J* = 9.0 Hz), 4.25 (1.2H, d, *J* = 11.3 Hz), 4.16 (1.4H, m), 3.97 (4.3H, m), 3.74 (3.7H, m), 3.56 (2.5H, bs), 3.12 (2.7H, bs), 3.04 (2.0H, bs), 2.64 (5.7H, bs), 2.16 (4.4H, bs), 2.04 (4.3H, s), 2.02 (4.8H, s), 1.95 (8.9H, s), 1.92 (7.3H, s), 1.84 (3.9H, s) ppm. MALDI-TOF (pos) *m*/*z* 24,400.

**Table 5:** MALDI-TOF MS data for heterogeneously functionalized dendrimers **4a**–**f**, **5a**–**f** and **6a**–**f**.

Compound Number	M_w_ after Galactose Addition	M_w_ after Lactose Addition	M_w_ after Deacylation
**4a**	6800	24,400	15,000
**4b**	10,200	24,600	15,100
**4c**	13,300	22,200	14,900
**4d**	15,800	21,800	15,700
**4e**	18,400	20,500	15,800
**4f**	19,500	6800	15,200
**5a**	13,500	54,500	31,200
**5b**	21,200	48,000	31,700
**5c**	26,700	45,400	33,000
**5d**	30,500	41,700	34,300
**5e**	35,500	41,000	34,300
**5f**	40,900	13,500	31,500
**6a**	51,000	147,000	100,000
**6b**	68,000	144,000	101,000
**6c**	78,500	140,500	101,500
**6d**	90,500	132,500	102,000
**6e**	102,000	131,500	106,500
**6f**	122,000	51,000	101,000

**4b**: ^1^H-NMR (500 MHz *d*_6_-DMSO) δ 7.95 (1H, bs), 7.75 (1.1H, bs), 7.47 (2H, bs), 5.21 (0.4H, s), 5.17 (1.3H, s), 5.12 (2.6H, m), 4.89 (0.4H, m), 4.79 (1.5H, app t, *J* = 9.0 Hz), 4.69 (2.4H, m), 4.62 (1.1H, *J* = 9.0 Hz), 4.25 (1.2H, d, *J* = 11.3Hz), 4.16 (1.7H, m), 3.98 (4.8H, m), 3.74 (4.1H, m), 3.40–3.56 (16H, m), 3.13 (3.2H, bs), 3.04 (2.3H, bs), 2.64 (5.9H, bs), 2.16 (4.2H, bs), 2.07 (1.2H, s), 2.05 (3.6H, s), 2.03 (3.1H, s), 1.96 (10.2H, s), 1.92 (6.2H, s), 1.87 (1.2H, s), 1.84 (3.9H, s) ppm. MALDI-TOF (pos) *m*/*z* 24,600.

**4c**: ^1^H-NMR (500 MHz *d*_6_-DMSO) δ 7.96 (1H, bs), 7.75 (1.1H, bs), 7.47 (1.9H, bs), 5.21 (0.6H, s), 5.17 (0.9H, s), 5.12 (1.8H, m), 4.89 (0.6H, app t, *J* = 9.1 Hz), 4.79 (0.9H, app t, *J* = 9.0 Hz), 4.69 (1.8H, m), 4.62 (0.6H, *J* = 9.0 Hz), 4.25 (0.7H, d, *J* = 11.3Hz), 4.17 (1.5H, m), 3.98 (3.8H, m), 3.74 (3.2H, m), 3.40–3.56 (20H, m), 3.13 (2.7H, bs), 3.04 (2.3H, bs), 2.64 (4.4H, bs), 2.37 (2.9H, bs), 2.16 (4.1H, bs), 2.07 (1.9H, s), 2.05 (2.1H, s), 2.03 (2.5H, s), 1.95 (8.4H, s), 1.92 (4.6H, s), 1.87 (1.5H, s), 1.85 (2.3H, s) ppm. MALDI-TOF (pos) *m*/*z* 22,200.

**4d**: ^1^H-NMR (500 MHz *d*_6_-DMSO) δ 7.96 (1H, bs), 7.75 (0.9H, bs), 7.47 (1.7H, bs), 5.21 (0.7H, d, *J* = 3.1 Hz), 5.17 (0.5H, s), 5.12 (1.5H, m), 4.89 (0.7H, app t, *J* = 9.1 Hz), 4.79 (0.5H, app t, *J* = 9.0 Hz), 4.69 (1.3H, m), 4.62 (0.4H, *J* = 9.0 Hz), 4.25 (0.4H, d, *J* = 11.3 Hz), 4.17 (1.2H, m), 3.98 (3H, m), 3.74 (2.3H, m), 3.40–3.56 (13H, m), 3.13 (2.5H, bs), 3.04 (1.8H, bs), 2.64 (3.8H, bs), 2.37 (2.1H, bs), 2.16 (3.5H, bs), 2.07 (2.2H, s), 2.05 (1.2H, s), 2.03 (2.3H, s), 1.95 (4.9H, s), 1.92 (2.2H, s), 1.87 (1.6H, s), 1.85 (1.3H, s) ppm. MALDI-TOF (pos) *m*/*z* 21,800.

**4e**: ^1^H-NMR (500 MHz *d*_6_-DMSO) δ 7.96 (1H, bs), 7.75 (0.9H, bs), 7.47 (1.7H, bs), 5.21 (0.9H, s), 5.17 (0.2H, s), 5.12 (1.3H, m), 4.89 (0.9H, app t, *J* = 9.1 Hz), 4.79 (0.2H, app t, *J* = 9.0 Hz), 4.69 (1.1H, m), 4.62 (0.2H, *J* = 9.0 Hz), 4.25 (0.2H, d, *J* = 11.3 Hz), 4.17 (1.2H, m), 3.98 (2.6H, m), 3.74 (1.9H, m), 3.40–3.56 (13H, m), 3.13 (2.7H, bs), 3.04 (1.7H, bs), 2.64 (5H, bs), 2.37 (3H, bs), 2.16 (3.6H, bs), 2.07 (2.7H, s), 2.05 (0.7H, s), 2.03 (0.6H, s), 1.95 (6.9H, s), 1.92 (1.4H, s), 1.87 (3H, s) ppm. MALDI-TOF (pos) *m*/*z* 20,500.

**4f**: ^1^H-NMR (500 MHz *d*_6_-DMSO) δ 7.95 (1H, bs, amide NH's), 7.74 (1H, bs, amide NH's), 7.46 (2H, bs, CH_2_N*H*C(S)N*H*CH_2_), 5.21 (1.3H, d, *J* = 3.2 Hz, H4), 5.11 (1.3H, dd, *J* = 3.2, 10.3 Hz, H3), 4.89 (1.3H, dd, *J* = 8.1, 10.3 Hz, H2), 4.69 (1.3H, d, *J* = 8.1 Hz, H1), 4.12 (1.3H, d, *J* = 6.1 Hz, OC*H*_2_CH_2_O), 4.01 (2.6H, m), 3.76 (1.3H, m), 3.59 (1.3H, m), 3.27–3.52 (12H, m), 3.13 (3H, bs), 3.04 (2.2H, bs), 2.61 (4.8H, m), 2.39 (3.2H, m), 2.16 (4.8H, bs), 2.08 (4H, s), 2.00 (4H, s), 1.96 (4H, s), 1.87 (4H, s) ppm. MALDI-TOF (pos) *m*/*z* 19,500.

**5a**: ^1^H-NMR (500 MHz *d*_6_-DMSO) δ 7.95 (1H, bs), 7.78 (0.8H, bs), 7.47 (1.9H, bs), 5.17 (1.7H, s), 5.11 (2.8H, m), 4.79 (1.7H, app t, *J* = 9.0 Hz), 4.69 (2.5H, m), 4.62 (1.2H, *J* = 9.0 Hz), 4.25 (1.4H, d, *J* = 10.8 Hz), 4.16 (1.6H, m), 3.97 (4.9H, m), 3.74 (4.4H, m), 3.40–3.56 (10H, m), 3.12 (2.7H, bs), 3.04 (1.4H, bs), 2.64 (4.9H, bs), 2.16 (3.8H, bs), 2.04 (5.2H, s), 2.02 (4.7H, s), 1.95 (10.5H, s), 1.92 (7.4H, s), 1.84 (4.6H, s) ppm. MALDI-TOF (pos) *m*/*z* 54,500.

**5b**: ^1^H-NMR (500 MHz *d*_6_-DMSO) δ 7.95 (1H, bs), 7.75 (1H, bs), 7.47 (1.7H, bs), 5.21 (0.3H, s), 5.17 (1.1H, s), 5.12 (2.1H, m), 4.89 (0.3H, m), 4.79 (1.1H, app t, *J* = 9.0 Hz), 4.69 (1.9H, m), 4.62 (0.8H, *J* = 9.0 Hz), 4.25 (1H, d, *J* = 11.3 Hz), 4.16 (1.3H, m), 3.98 (3.8H, m), 3.74 (3.2H, m), 3.40–3.56 (10H, m), 3.13 (2.7H, bs), 3.04 (2.1H, bs), 2.64 (5.7H, bs), 2.16 (3.9H, bs), 2.07 (1.1H, s), 2.05 (2.7H, s), 2.03 (3H, s), 1.96 (8.2H, s), 1.92 (4.5H, s), 1.87 (1H, s), 1.84 (2.5H, s) ppm. MALDI-TOF (pos) *m*/*z* 48,000.

**5c**: ^1^H-NMR (500 MHz *d*_6_-DMSO) δ 7.96 (1H, bs), 7.75 (1H, bs), 7.46 (1.7H, bs), 5.21 (0.5H, s), 5.17 (0.8H, s), 5.12 (1.8H, m), 4.89 (0.5H, app t, *J* = 9.1 Hz), 4.79 (0.8H, app t, *J* = 9.0 Hz), 4.69 (1.6H, m), 4.62 (0.6H, *J* = 9.0 Hz), 4.25 (0.7H, d, *J* = 11.3 Hz), 4.17 (1.3H, m), 3.98 (3.5H, m), 3.74 (2.8H, m), 3.40–3.56 (10H, m), 3.13 (2.5H, bs), 3.04 (2H, bs), 2.64 (4.9H, bs), 2.37 (2.3H, bs), 2.16 (4H, bs), 2.07 (1.6H, s), 2.05 (2.2H, s), 2.03 (1.8H, s), 1.95 (7.8H, s), 1.92 (3.5H, s), 1.87 (1.4H, s), 1.85 (1.8H, s) ppm. MALDI-TOF (pos) *m*/*z* 45,500.

**5d**: ^1^H-NMR (500 MHz *d*_6_-DMSO) δ 7.96 (1H, bs), 7.75 (0.9H, bs), 7.47 (1.9H, bs), 5.21 (0.8H, d, *J* = 3.1 Hz), 5.17 (0.6H, s), 5.12 (1.7H, m), 4.89 (0.7H, app t, *J* = 9.1 Hz), 4.79 (0.5H, app t, *J* = 9.0 Hz), 4.69 (1.5H, m), 4.62 (0.4H, *J* = 9.0 Hz), 4.25 (0.4H, d, *J* = 11.3 Hz), 4.17 (1.4H, m), 3.98 (3.2H, m), 3.74 (2.6H, m), 3.40–3.56 (10H, m), 3.13 (2.6H, bs), 3.04 (2.1H, bs), 2.64 (6.2H, bs), 2.37 (3H, bs), 2.16 (3.9H, bs), 2.07 (2.2H, s), 2.05 (1.4H, s), 2.03 (1.2H, s), 1.95 (8.3H, s), 1.92 (2.5H, s), 1.87 (1.9H, s), 1.85 (1.5H, s) ppm. MALDI-TOF (pos) *m*/*z* 41,500.

**5e**: ^1^H-NMR (500 MHz *d*_6_-DMSO) δ 7.96 (1H, bs), 7.75 (0.9H, bs), 7.47 (2H, bs), 5.21 (1H, s), 5.17 (0.3H, s), 5.12 (1.5H, m), 4.89 (1H, app t, *J* = 9.1 Hz), 4.79 (0.2H, app t, *J* = 9.0 Hz), 4.69 (1H, m), 4.62 (0.2H, *J* = 9.0 Hz), 4.25 (0.3H, d, *J* = 11.3 Hz), 4.17 (1H, m), 3.98 (2.9H, m), 3.74 (2.1H, m), 3.40–3.56 (12H, m), 3.13 (2.7H, bs), 3.04 (1.9H, bs), 2.64 (5.2H, bs), 2.37 (2.8H, bs), 2.16 (4.3H, bs), 2.07 (2.8H, s), 2.05 (1H, s), 2.03 (0.9H, s), 1.95 (8.8H, s), 1.92 (1.5H, s), 1.87 (2.5H, s) ppm. MALDI-TOF (pos) *m*/*z* 41000.

**5f**: ^1^H-NMR (500 MHz *d*_6_-DMSO) δ 7.94 (1H, bs, amide NH's), 7.73 (1H, bs, amide NH's), 7.45 (2H, bs, CH_2_N*H*C(S)N*H*CH_2_), 5.21 (1.3H, s, H4), 5.11 (1.3H, d, *J* = 10.3 Hz, H3), 4.89 (1.3H, m, H2), 4.69 (1.3H, m, H1), 4.13 (1.3H, s, OC*H*_2_CH_2_O), 4.01 (2.7H, m), 3.76 (1.7H, m), 3.27–3.52 (12H, m), 3.13 (3H, bs), 3.04 (2.0H, bs), 2.61 (4.5H, m), 2.39 (2.7H, m), 2.16 (4.2H, bs), 2.08 (4.1H, s), 2.00 (3.1H, s), 1.96 (4.6H, s), 1.87 (3.7H, s) ppm. MALDI-TOF (pos) *m*/*z* 40,900.

**6a**: ^1^H-NMR (500 MHz *d*_6_-DMSO) δ 7.96 (1H, bs), 7.76 (0.7H, bs), 7.47 (1.5H, bs), 5.17 (1H, s), 5.11 (1.8H, m), 4.79 (1.2H, m), 4.69 (1.5H, m), 4.62 (0.9H, m), 4.25 (0.9H, m), 4.16 (1H, m), 3.97 (3.2H, m), 3.74 (2.9H, m), 3.56 (1.6H, bs), 3.04–3.12 (3.8H, m), 2.64 (2.4H, bs), 2.16 (3.3H, bs), 2.04 (3H, s), 2.02 (3.4H, s), 1.95 (7.1H, s), 1.92 (4.9H, s), 1.84 (3.1H, s) ppm. MALDI-TOF (pos) *m*/*z* 147,000.

**6b**: ^1^H-NMR (500 MHz *d*_6_-DMSO) δ 7.95 (1H, bs), 7.75 (0.8H, bs), 7.47 (1.6H, bs), 5.21 (0.3H, s), 5.17 (0.8H, s), 5.12 (1.8H, m), 4.89 (0.2H, m), 4.79 (1.2H, m), 4.69 (1.6H, m), 4.62 (0.8H, m), 4.25 (0.8H, m), 4.16 (1.2H, m), 3.98 (3.4H, m), 3.74 (2.8H, m), 3.40–3.56 (15H, m), 3.13 (2.6H, bs), 3.04 (1.6H, bs), 2.16 (3.6H, bs), 2.05 (4.3H, s), 2.03 (3.5H, s), 1.96 (8H, s), 1.92 (4.8H, s), 1.84 (3.8H, s) ppm. MALDI-TOF (pos) *m*/*z* 144,000.

**6c**: ^1^H-NMR (500 MHz *d*_6_-DMSO) δ 7.96 (1H, bs), 7.75 (0.8H, bs), 7.47 (1.4H, bs), 5.21 (0.4H, s), 5.17 (0.6H, s), 5.12 (1.4H, m), 4.89 (0.3H, m), 4.79 (0.7H, m), 4.69 (1.2H, m), 4.62 (0.5H, m), 4.25 (0.5H, m), 4.17 (1H, m), 3.98 (2.6H, m), 3.74 (2H, m), 3.40–3.56 (8H, m), 3.13 (2H, bs), 3.04 (1.6H, bs), 2.64 (3.9H, bs), 2.37 (2.3H, bs), 2.16 (2.9H, bs), 2.07 (1.2H, s), 2.05 (1.6H, s), 2.03 (2.3H, s), 1.95 (6.1H, s), 1.92 (3.1H, s), 1.87 (1H, s), 1.85 (1.9H, s) ppm. MALDI-TOF (pos) *m*/*z* 140,500.

**6d**: ^1^H-NMR (500 MHz *d*_6_-DMSO) δ 7.96 (1H, bs), 7.75 (0.8H, bs), 7.47 (1.6H, bs), 5.21 (0.6H, m), 5.17 (0.4H, s), 5.12 (1.3H, m), 4.89 (0.5H, m), 4.79 (0.5H, m), 4.69 (1.1H, m), 4.62 (0.4H, m), 4.25 (0.4H, m), 4.17 (1H, m), 3.98 (2.4H, m), 3.74 (1.8H, m), 3.40–3.56 (8.6H, m), 3.13 (2.4H, bs), 3.04 (1.6H, bs), 2.64 (4.1H, bs), 2.37 (1.9H, bs), 2.16 (3.1H, bs), 2.07 (1.7H, s), 2.05 (1.2H, s), 2.03 (1.6H, s), 1.95 (6H, s), 1.92 (2.3H, s), 1.87 (1.3H, s), 1.85 (1.1H, s) ppm. MALDI-TOF (pos) *m*/*z* 132,500.

**6e**: ^1^H-NMR (500 MHz *d*_6_-DMSO) δ 7.96 (1H, bs), 7.75 (0.8H, bs), 7.47 (1.6H, bs), 5.21 (0.8H, s), 5.17 (0.3H, s), 5.12 (1.2H, m), 4.89 (0.7H, m), 4.79 (0.3H, m), 4.69 (1.1H, m), 4.62 (0.2H, m), 4.25 (0.2H, m), 4.17 (1.1H, m), 3.98 (2.5H, m), 3.74 (1.5H, m), 3.40–3.56 (9H, m), 3.13 (2.4H, bs), 3.04 (1.7H, bs), 2.64 (4.8H, bs), 2.37 (2.9H, bs), 2.16 (3.2H, bs), 2.07 (2.3H, s), 2.05 (0.8H, s), 2.03 (1.3H, s), 1.95 (6.8H, s), 1.92 (1.7H, s), 1.87 (2.1H, s), 1.85 (1H, s) ppm. MALDI-TOF (pos) *m*/*z* 131,500.

**6f**: ^1^H-NMR (500 MHz *d*_6_-DMSO) δ 7.95 (1H, bs, amide NH's), 7.74 (0.9H, bs, amide NH's), 7.45 (2H, bs, CH_2_N*H*C(S)N*H*CH_2_), 5.21 (1.2H, s, H4), 5.11 (1.3H, d, *J* = 10.3 Hz, H3), 4.89 (1.3H, m, H2), 4.69 (1.1H, d, *J* = 8.1 Hz, H1), 4.12 (1.3H, d, *J* = 6.1 Hz, OC*H*_2_CH_2_O), 4.01 (2.7H, m), 3.76 (1.9H, m), 3.27–3.59 (11.8H, m), 3.13 (3H, bs), 3.04 (2.2H, bs), 2.61 (4.5H, m), 2.39 (1.8H, m), 2.16 (3.9H, bs), 2.08 (3.9H, s), 2.00 (8.4H, s), 1.87 (3.6H, s) ppm. MALDI-TOF (pos) *m*/*z* 122,000.

##### Deacetylated

**4a**: ^1^H-NMR (500 MHz *d*_6_-DMSO) δ 8.02 (bs, 1H), 7.82 (bs 0.9H), 7.54 (bs, 1.4H), 5.12 (bm, 1.8H), 4.64 (m, 3.7H), 4.33 (m, 2.1H), 3.87 (d, *J* = 4.8 Hz, 1.4H), 3.74 (m, 1.7H), 3.47–3.62 (m, 18H), 3.17 (bs, 2.6H), 3.08 (bs, 1.7H), 3.03 (m, 1.3H), 2.66 (bs, 3.7H), 2.43 (bs, 1.9H), 2.20 (bs, 3.6H), 1.89 (s, 0.4H), 1.80 (s, 0.2H) ppm. MALDI-TOF (pos) *m*/*z* 15,000.

**4b**: ^1^H-NMR (500 MHz *d*_6_-DMSO) δ 8.04 (bs, 1H), 7.51 (bs 0.8H), 5.08 (bm, 0.8H), 4.53–4.70 (m, 2.6H), 4.20 (m, 1.0H), 4.09 (s, 0.2H), 3.83 (bs, 0.9H), 3.77 (m, 1.0H), 3.43–3.58 (m, 13H), 3.14 (bs, 2.0H), 3.05 (bs, 0.6H), 2.62 (bs, 1.8H), 2.39 (bs, 1.9H), 2.26 (bs, 1.6H), 1.85 (s, 0.1H), 1.76 (s, 0.2H) ppm MALDI-TOF (pos) *m*/*z* 15,100.

**4c**: ^1^H-NMR (500 MHz *d*_6_-DMSO) δ 8.03 (bs, 1H), 7.51 (bs 0.8H), 5.08 (bm, 0.6H), 4.53–4.70 (m, 2.1H), 4.20 (m, 0.6H), 4.09 (s, 0.3H), 3.82 (bs, 0.7H), 3.72 (m, 0.7H), 3.43–3.58 (m, 14H), 3.14 (bs, 1.8H), 2.99 (bs, 0.7H), 2.73 (bs, 1.8H), 2.24 (bs, 1.8H), 1.85 (s, 0.1H), 1.76 (s, 0.3H) ppm. MALDI-TOF (pos) *m*/*z* 14,900.

**4d**: ^1^H-NMR (500 MHz *d*_6_-DMSO) δ 8.10 (bs, 1H), 7.55 (bs 0.9H), 5.08 (bm, 0.5H), 4.53–4.70 (m, 2.2H), 4.23 (m, 0.6H), 4.12 (s, 0.6H), 3.85 (bs, 1.2H), 3.72 (m, 0.7H), 3.43–3.58 (m, 15H), 3.14 (bs, 1.8H), 2.84 (bs, 1.9H), 2.64 (bs, 1.0H), 2.32 (bs, 1.9H), 1.85 (s, 0.1H), 1.76 (s, 0.1H) ppm. MALDI-TOF (pos) *m*/*z* 15,700.

**4e**: ^1^H-NMR (500 MHz *d*_6_-DMSO) δ 8.08 (bs, 1H), 7.51 (bs 0.9H), 4.33–5.30 (m, 2.5H), 4.22 (m, 0.4H), 4.12 (s, 0.9H), 3.82 (bs, 1.5H), 3.43–3.65 (m, 20H), 3.14 (bs, 2.3H), 2.82 (bs, 2.2H), 2.62 (bs, 0.8H), 2.24 (bs, 2.0H) ppm. MALDI-TOF (pos) *m*/*z* 15,800.

**4f**: ^1^H-NMR (500 MHz *d*_6_-DMSO) δ 7.99 (bs, 1H), 7.80 (bs, 1H), 7.50 (bs, 2.1H), 4.96 (bs, 1.0H), 4.73 (m, 1.0H), 4.61 (bs, 1.2H), 4.44 (bs, 1.2H), 4.10 (d, *J* = 5.2 Hz, 1.2H), 3.84 (m, 1.5H), 3.63 (m, 2.4H), 3.38–3.60 (m, 30H), 3.17 (bs, 3.2H), 3.08 (bs, 3.2H), 2.66 (bs, 4.6H), 2.42 (bs, 2.4H), 2.20 (bs, 4.6H) ppm. MALDI-TOF (pos) *m*/*z* 15,200.

**5a**: ^1^H-NMR (500 MHz *d*_6_-DMSO) δ 8.03 (bs, 1H), 7.86 (bs 0.8H), 7.52 (bs, 1.7H), 5.22 (bs, 1.0H), 5.12 (bm, 1.1H), 4.82 (bs 1.1H), 4.72 (m, 2.1H), 4.55 (m, 1.9H), 4.22 (m, 2.1H), 3.87 (d, 1.4H), 3.74 (m, 1.8H), 3.47–3.62 (m, 34H), 3.17 (bs, 2.2H), 3.03 (bs, 1.2H), 2.70 (bs, 3.1H), 2.23 (bs, 3.2H) ppm. MALDI-TOF (pos) *m*/*z* 31,200.

**5b**: ^1^H-NMR (500 MHz *d*_6_-DMSO) δ 8.11 (bs, 1H), 7.57 (bs 0.9H), 5.13 (bm, 0.9H), 4.53–4.70 (m, 2.3H), 4.22 (m, 0.9H), 4.11 (s, 0.2H), 3.86 (bs, 0.9H), 3.76 (m, 0.8H), 3.43–3.58 (m, 18H), 3.14 (bs, 1.7H), 3.05 (bs, 0.8H), 2.85 (bs, 1.6H), 2.39 (bs, 1.5H) ppm. MALDI-TOF (pos) *m*/*z* 31,700.

**5c**: ^1^H-NMR (500 MHz *d*_6_-DMSO) δ 8.06 (bs, 1H), 7.95 (bs, 0.7H), 7.54 (bs 1.3H), 5.11 (bs, 0.9H), 4.53–4.70 (m, 3.2H), 4.20 (m, 1.0H), 4.09 (s, 0.4H), 3.82 (bs, 1.1H), 3.72 (m, 0.9H), 3.43–3.58 (m, 29H), 3.14 (bs, 3.3H), 3.03 (bs, 0.9H), 2.77 (bs, 3.0H), 2.24 (bs, 3.1H) ppm. MALDI-TOF (pos) *m*/*z* 33,000.

**5d**: ^1^H-NMR (500 MHz *d*_6_-DMSO) δ 8.06 (bs, 1H), 7.95 (bs, 0.7H), 7.55 (bs 1.3H), 4.53–5.30 (m, 4.0H), 4.22 (m, 0.8H), 4.12 (s, 0.8H), 3.85 (bs, 1.4H), 3.72 (m, 0.7H), 3.43–3.58 (m, 21H), 3.14 (bs, 3.5H), 2.84 (bs, 3.2H), 2.64 (bs, 0.8H), 2.32 (bs, 2.7H) ppm. MALDI-TOF (pos) *m*/*z* 34,300.

**5e**: ^1^H-NMR (500 MHz *d*_6_-DMSO) δ 8.02 (bs, 1H), 7.86 (bs, 0.9H), 7.51 (bs, 1.7H), 4.33–5.30 (m, 4.0H), 4.22 (m, 0.3H), 4.12 (s, 1.0H), 3.85 (bs, 1.3H), 3.43–3.65 (m, 29H), 3.18 (bs, 4,4H), 2.70 (bs, 3.8H), 2.23 (bs, 3.7H) ppm. MALDI-TOF (pos) *m*/*z* 34,300.

**5f**: ^1^H-NMR (500 MHz *d*_6_-DMSO) δ 7.97 (bs, 1H), 7.78 (bs, 0.9H), 7.44 (bs, 2.0H), 4.97 (bs, 0.8H), 4.75 (m, 0.9H), 4.65 (bs, 1.2H), 4.44 (bs, 1.2H), 4.08 (m, 1.2H), 3.80 (m, 1.5H), 3.38–3.65 (m, 30H), 3.13 (bs, 2.9H), 3.05 (bs, 2.3H), 2.62 (bs, 4.3H), 2.46 (bs, 2.1H), 2.17 (bs, 3.9H) ppm. MALDI-TOF (pos) *m*/*z* 31,500.

**6a**: ^1^H-NMR (500 MHz *d*_6_-DMSO) δ 8.02 (bs, 1H), 7.81 (bs 0.9H), 7.53 (bs, 1.3H), 5.22 (bs, 0.8H), 5.12 (bs, 0.8H), 4.64 (m, 3.7H), 4.22 (m, 1.7H), 3.87 (m, 1.2H), 3.74 (bs, 1.4H), 3.47–3.62 (m, 28H), 3.17 (bs, 1.8H), 3.08 (bs, 2.4H), 2.66 (bs, 2.7H), 2.43 (bs, 1.3H), 2.20 (bs, 3.0H) ppm. MALDI-TOF (pos) *m*/*z* 100,000.

**6b**: ^1^H-NMR (500 MHz *d*_6_-DMSO) δ 8.08 (bs, 1H), 7.57 (bs 0.8H), 5.15 (bm, 0.9H), 4.53–4.79 (m, 2.3H), 4.23 (m, 0.9H), 4.10 (s, 0.2H), 3.83 (bs, 0.9H), 3.77 (m, 1.0H), 3.43–3.58 (m, 16H), 3.18 (bs, 1.0H), 3.03 (bs, 0.9H), 2.77 (bs, 1.2H), 2.29 (bs, 1.0H) ppm. MALDI-TOF (pos) *m*/*z* 101,000.

**6c**: ^1^H-NMR (500 MHz *d*_6_-DMSO) δ 8.06 (bs, 1H),7.83 (bs, 0.6H), 7.55 (bs 1.2H), 5.13 (m 1.2H), 4.53–4.70 (m, 3.1H), 4.23 (m, 1.0H), 4.12 (s, 0.4H), 3.86 (bs, 1.4H), 3.72 (m, 1.0H), 3.43–3.58 (m, 21H), 3.18 (bs, 1.8H), 3.03 (bs, 0.8H), 2.73 (bs, 2.6H), 2.25 (bs, 2.3H) ppm. MALDI-TOF (pos) *m*/*z* 101,500.

**6d**: ^1^H-NMR (500 MHz *d*_6_-DMSO) δ 8.03 (bs, 1H), 7.89 (bs, 0.9H), 7.55 (bs, 1.5H), 5.13 (m, 0.9H), 4.53–4.70 (m, 3.1H), 4.23 (m, 0.8H), 4.12 (s, 0.7H), 3.85 (bs, 1.2H), 3.72 (m, 0.4H), 3.43–3.58 (m, 23H), 3.14 (bs, 4.5H), 2.70 (bs, 3.5H), 2.23 (bs, 3.5H) ppm. MALDI-TOF (pos) *m*/*z* 102,000.

**6e**: ^1^H-NMR (500 MHz *d*_6_-DMSO) δ 8.06 (bs, 1H), 7.55 (bs 0.8H), 4.33–5.30 (m, 2.2H), 4.22 (m, 0.4H), 4.12 (s, 0.5H), 3.82 (bs, 0.8H), 3.43–3.65 (m, 12H), 3.14 (bs, 2.2H), 2.77 (bs, 1.7H), 2.28 (bs, 1.8H) ppm. MALDI-TOF (pos) *m*/*z* 106,500.

**6f**: ^1^H-NMR (500 MHz *d*_6_-DMSO) δ 8.00 (bs, 1H), 7.80 (bs, 0.9H), 7.48 (bs, 1.6H), 4.99 (bs, 0.7H), 4.80 (m, 0.7H), 4.63 (bs, 0.9H), 4.48 (bs, 0.8H), 4.12 (s, 0.8H), 3.80 (m, 0.9H), 3.38–3.65 (m, 14H), 3.18 (bs, 2.4H), 3.09 (bs, 1.7H), 2.62 (bs, 3.3H), 2.46 (bs, 1.5H), 2.17 (bs, 4.0H) ppm. MALDI-TOF (pos) *m*/*z* 101,000.

#### 3.5.5. Representative Procedure for the Synthesis of Acetyl Protected N-Acetylgalactose and Galactose Functionalized Dendrimers

An aqueous solution of amine terminated Starburst G(4)-PAMAM dendrimer (2.478 g of a 17% w/w solution in water, 421.2 mg, 31.2 μmol) was lyophilized to leave a foamy residue. DMSO (7.02 mL) was added to this residue to give a 60 mg/mL solution. A 300 mM solution of **3** (0.35 mL, 85 μmol, 40.2 mg) in DMSO was added to the 60 mg/mL G(4) PAMAM dendrimer solution (0.5 mL, 30 mg, 4.40 μmol). The reaction was stirred for 8 h at which point a 75 μL aliquot was removed for MALDI-TOF analysis. After MALDI-TOF analysis indicated reaction completion, a 300 mM solution of **1** (0.31 mL, 73 μmol, 35 mg) was added. The solution was then stirred for 8 h, lyophilized, and directly subjected to deacetylation conditions. The amounts of reagents used for individual compounds are listed in Table 6.

**Table 6:** Amounts used in synthesis of compounds **7a**–**7f**, **8a**–**8f** and **9a**–**9f**.

Compound Number	PAMAM Generation	PAMAM (μmol)	GalNAc (μmol) (q ^a^)	Galactose (μmol) (r ^a^)
**7a**	3	4.4	0	140.8
**7b**	3	4.4	28.2	112.6
**7c**	3	4.4	56.3	84.5
**7d**	3	4.4	84.5	56.3
**7e**	3	4.4	112.6	28.2
**7f**	3	4.4	140.8	0
**8a**	4	2.2	0	140.8
**8b**	4	2.2	28.2	112.6
**8c**	4	2.2	56.3	84.5
**8d**	4	2.2	84.5	56.3
**8e**	4	2.2	112.6	28.2
**8f**	4	2.2	140.8	0
**9a**	6	0.5	0	120.0
**9b**	6	0.5	24.0	96.0
**9c**	6	0.5	48.0	72.0
**9d**	6	0.5	72.0	48.0
**9e**	6	0.5	96.0	24.0
**9f**	6	0.5	120.0	0

^a^ Values for q and r (as shown in Scheme 2 of the primary manuscript) derive directly from the μmol values shown here.

#### 3.5.6. General Deacetylation Procedure to Afford Dendrimers **7**–**9**

To the lyophilized solid peracetylated dendrimers, 1 mL of 1:1 water-methanol was added, at which point the dendrimer became a white precipitate. To this mixture was added NaOMe (0.2 equiv., 0.8 M in MeOH) for each peripheral carbohydrate, and it was allowed to stir for 3 h. If, at this time, the mixture had not become a clear solution a further 0.2 equivalents of NaOMe (0.8 M in MeOH) was added, and this step was repeated until the mixture became a clear and colorless solution. Aqueous HCl solution (0.1 M) was then added slowly until the pH was ~7. This neutralized solution was placed in a dialysis membrane (Mw cutoff 3,500 Da) and dialyzed in 1 L of DI water for 8 h. The water was changed and let stand for a further 8 h twice more. The remaining liquid in the membrane was frozen and lyophilized to give a white fluffy solid. Compounds were characterized with ^1^H-NMR and MALDI-TOF MS. MALDI-TOF MS data is summarized in Table 7.

**Table 7:** MALDI-TOF MS data for heterogeneously functionalized dendrimers **7a**–**f**, **8a**–**f** and **9a**–**f**.

Compound Number	M_w_ after Galactose Addition	M_w_ after Galactosamine Addition	M_w_ after Deacylation
**7a**	19100	8950	15000
**7b**	19000	11700	15100
**7c**	18900	13900	14900
**7d**	19300	15700	15700
**7e**	19000	18200	15800
**7f**	n/a *	20000	16400
**8a**	40000	18200	31200
**8b**	39700	23900	31700
**8c**	40200	28300	33000
**8d**	39700	33500	34300
**8e**	40500	37200	34300
**8f**	n/a	39300	33300
**9a**	119500	66500	100000
**9b**	119000	77500	101000
**9c**	119000	92000	101500
**9d**	121500	101000	102000
**9e**	121500	117000	106500
**9f**	n/a	125000	107500

* n/a = not available.

#### 3.5.7. Spectroscopic Data for Compounds **7**–**9**

##### Acetylated

**7a**: ^1^H-NMR (500 MHz *d*_6_-DMSO) δ 7.95 (1H, bs, amide NH's), 7.74–7.79 (1.4H, m, amide NH's, N*H*'Ac), 7.46 (2H, bs, CH_2_N*H*C(S)N*H*CH_2_), 5.21 (1.2H, d, *J* = 3.2 Hz, H4), 5.17 (0.2H, s, H4'), 5.11 (1.2H, dd, *J* = 3.2, 10.3 Hz, H3), 4.92 (0.2H, d, *J* = 10.3 Hz, H3'), 4.89 (1.2H, t, *J* = 9.7 Hz, H2), 4.69 (1.2H, d, *J* = 7.6 Hz, H1), 4.51 (0.2H, d, *J* = 8.4 Hz, H1'), 4.12 (1.2H, m), 4.01 (3.1H, m), 3.83 (0.2H, app q, *J* = 8.4, 9.0, 10.3 Hz, H2'), 3.76 (2.0H, m), 3.27–3.59 (14.2H, m), 3.13 (3.1H, bs), 3.04 (2.4H, bs), 2.61 (3.1H, m), 2.16 (4.7H, bs), 2.08 (4H, s), 2.00 (2.8H, s), 1.96 (4.6H, s), 1.87 (3H, s) 1.72 (0.8H, s) ppm. MALDI-TOF (pos) *m*/*z* 19,100.

**7b**: ^1^H-NMR (500 MHz *d*_6_-DMSO) δ 7.95 (1H, bs, amide NH's), 7.79 (0.7H, d, *J* = 9.0 Hz, N*H*'Ac), 7.74 (0.9H, bs, amide NH's), 7.46 (2.1H, bs, CH_2_N*H*C(S)N*H*CH_2_), 5.21 (1.1H, d, *J* = 3.2 Hz, H4), 5.17 (0.4H, s, H4'), 5.11 (1.1H, dd, *J* = 3.2, 10.3 Hz, H3), 4.92 (0.4H, d, *J* = 10.3 Hz, H3'), 4.89 (1.1H, t, *J* = 9.7 Hz, H2), 4.69 (1.1H, d, *J* = 7.6 Hz, H1), 4.51 (0.4H, d, *J* = 8.4 Hz, H1'), 4.12 (1.1H, m), 4.01 (3.7H, m), 3.83 (0.4H, app q, *J* = 8.4, 9.0, 10.3 Hz, H2'), 3.76 (2.3H, m), 3.27–3.59 (15.9H, m), 3.13 (3.1H, bs), 3.04 (2.6H, bs), 2.61 (5.4H, m), 2.39 (3H, m), 2.16 (4.9H, bs), 2.08 (3H, s), 2.06 (1.5H, s), 1.96 (4.9H, s), 1.87 (2.9H, s), 1.85 (1.4H, s), 1.74 (1.2H, s) ppm. MALDI-TOF (pos) *m*/*z* 19,000.

**7c**: ^1^H-NMR (500 MHz *d*_6_-DMSO) δ 7.95 (1H, bs, amide NH's), 7.79 (0.8H, d, *J* = 9.0 Hz, N*H*'Ac), 7.74 (1H, bs, amide NH's), 7.46 (2H, bs, CH_2_N*H*C(S)N*H*CH_2_), 5.21 (0.8H, d, *J* = 3.2 Hz, H4), 5.17 (0.7H, s, H4'), 5.11 (0.8H, dd, *J* = 3.2, 10.3 Hz, H3), 4.92 (0.7H, d, *J* = 10.3 Hz, H3'), 4.89 (1.3H, t, *J* = 9.7 Hz, H2), 4.69 (1.3H, d, *J* = 7.6 Hz, H1), 4.51 (0.7H, d, *J* = 8.4 Hz, H1'), 4.12 (0.8H, m), 4.01 (3.8H, m), 3.83 (0.7H, app q, *J* = 8.4, 9.0, 10.3 Hz, H2'), 3.76 (1.9H, m), 3.27–3.59 (15.6H, m), 3.13 (3.2H, bs), 3.04 (2.6H, bs), 2.61 (5.6H, m), 2.39 (3H, m), 2.16 (4.7H, bs), 2.08 (4H, s), 2.00 (4H, s), 1.96 (4H, s), 1.87 (4H, s) ppm. MALDI-TOF (pos) *m*/*z* 18,900.

**7d**: ^1^H-NMR (500 MHz *d*_6_-DMSO) δ 7.95 (1H, bs, amide NH's), 7.79 (1.1H, d, *J* = 9.0 Hz, N*H*'Ac), 7.74 (0.9H, bs, amide NH's), 7.46 (2.1H, bs, CH_2_N*H*C(S)N*H*CH_2_), 5.21 (0.6H, d, *J* = 3.2 Hz, H4), 5.17 (0.9H, s, H4'), 5.11 (0.6H, dd, *J* = 3.2, 10.3 Hz, H3), 4.92 (0.9H, d, *J* = 10.3 Hz, H3'), 4.89 (0.6H, t, *J* = 9.7 Hz, H2), 4.69 (0.6H, d, *J* = 7.6 Hz, H1), 4.51 (0.9H, d, *J* = 8.4 Hz, H1'), 4.12 (0.6H, m), 4.01 (4.2H, m), 3.83 (0.9H, app q, *J* = 8.4, 9.0, 10.3 Hz, H2'), 3.76 (2.1H, m), 3.27–3.59 (15.7H, m), 3.13 (3.1H, bs), 3.04 (2.4H, bs), 2.61 (5.2H, m), 2.39 (3.1H, m), 2.16 (4.8H, bs), 2.07 (1.9H, s), 2.06 (2.8H, s), 1.98 (0.7H, s), 1.96 (4.9H, s), 1.87 (1.9H, s), 1.85 (2.5H, s), 1.74 (2.5H, s) ppm. MALDI-TOF (pos) *m*/*z* 19,300.

**7e**: ^1^H-NMR (500 MHz *d*_6_-DMSO) δ 7.95 (1H, bs, amide NH's), 7.79 (1.3H, d, *J* = 9.0 Hz, N*H*'Ac), 7.74 (0.8H, bs, amide NH's), 7.46 (2.1H, bs, CH_2_N*H*C(S)N*H*CH_2_), 5.21 (0.3H, d, *J* = 3.2 Hz, H4), 5.17 (1.1H, s, H4'), 5.11 (0.3H, dd, *J* = 3.2, 10.3 Hz, H3), 4.92 (1.1H, d, *J* = 10.3 Hz, H3'), 4.89 (0.3H, t, *J* = 9.7 Hz, H2), 4.69 (0.3H, d, *J* = 7.6 Hz, H1), 4.51 (1.1H, d, *J* = 8.4 Hz, H1'), 4.12 (0.8H, m), 4.01 (3.8H, m), 3.83 (1.1H, app q, *J* = 8.4, 9.0, 10.3 Hz, H2'), 3.76 (0.3H, m), 3.27–3.59 (15.3H, m), 3.13 (2.8H, bs), 3.04 (2.2H, bs), 2.61 (4.7H, m), 2.39 (2.7H, m), 2.16 (4.3H, bs), 2.07 (1.4H, s), 2.06 (3.4H, s), 1.95 (5H, s), 1.87 (1H, s), 1.85 (3.5H, s), 1.74 (3.3H, s) ppm. MALDI-TOF (pos) *m*/*z* 19,000.

**7f**: ^1^H-NMR (500 MHz *d*_6_-DMSO) δ 7.96 (1H, bs, amide NH's), 7.79 (1.3H, d, *J* = 9.0 Hz, N*H*'Ac), 7.74 (1.1H, bs, amide NH's), 7.46 (2.2H, m, CH_2_N*H*C(S)N*H*CH_2_), 5.17 (1.3H, s, H4'), 4.92 (1.3H, dd, *J* = 3.0, 10.3 Hz, H3'), 4.51 (1.3H, d, *J* = 8.4 Hz, H1'), 4.01 (4.7H, m), 3.83 (1.3H, app q, *J* = 8.4, 9.0, 10.3 Hz, H2'), 3.27–3.59 (16.5H, m), 3.13 (3.2H, bs), 3.04 (2.3H, bs), 2.61 (5.1H, m), 2.39 (2.5H, m), 2.16 (4.1H, bs), 2.06 (4.7H, s), 1.95 (4.7H, s), 1.85 (4.2H, s), 1.74 (4.2H, s) ppm. MALDI-TOF (pos) *m*/*z* 19,900.

**8a**: ^1^H-NMR (500 MHz *d*_6_-DMSO) δ 7.95 (1H, bs, amide NH's), 7.74–7.79 (1.4H, m, amide NH's, N*H*'Ac), 7.45 (2.1H, bs, CH_2_N*H*C(S)N*H*CH_2_), 5.21 (1.3H, s, H4), 5.17 (0.2H, s, H4'), 5.12 (1.3H, d, 10.5 Hz, H3), 4.92 (0.2H, m, H3'), 4.89 (1.3H, t, *J* = 9.7 Hz, H2), 4.69 (1.2H, d, *J* = 7.6 Hz, H1), 4.51 (0.2H, d, *J* = 8.4 Hz, H1'), 4.12 (1.3H, m), 4.00 (3.3H, m), 3.83 (0.2H, app q, *J* = 8.4, 9.0, 10.3 Hz, H2'), 3.76 (2.2H, m), 3.27–3.59 (12.7H, m), 3.13 (2.8H, bs), 3.04 (2.4H, bs), 2.61 (5.1H, m), 2.16 (4.8H, bs), 2.08 (4.7H, s), 2.00 (2.5H, s), 1.96 (5.3H, s), 1.87 (3.3H, s) 1.72 (0.8H, s) ppm. MALDI-TOF (pos) *m*/*z* 40,000.

**8b**: ^1^H-NMR (500 MHz *d*_6_-DMSO) δ 7.95 (1H, bs, amide NH's), 7.79 (0.7H, d, *J* = 9.0 Hz, N*H*'Ac), 7.74 (1.0H, bs, amide NH's), 7.46 (2.1H, m, CH_2_N*H*C(S)N*H*CH_2_), 5.21 (1.2H, s, H4), 5.17 (0.5H, s, H4'), 5.11 (1.3H, m, H3), 4.92 (0.5H, d, *J* = 10.3 Hz, H3'), 4.89 (1.3H, t, *J* = 9.7 Hz, H2), 4.69 (1.1H, d, *J* = 7.6 Hz, H1), 4.51 (0.4H, d, *J* = 8.4 Hz, H1'), 4.12 (1.3H, m), 4.01 (4.1H, m), 3.83 (0.6H, app q, *J* = 8.4, 9.0, 10.3 Hz, H2'), 3.76 (2.9H, m), 3.27–3.59 (13.8H, m), 3.13 (3.0H, bs), 3.04 (2.6H, bs), 2.61 (4.4H, m), 2.39 (2.7H, m), 2.16 (4.4H, bs), 2.08 (3.6H, s), 2.06 (1.6H, s), 1.96 (5.9H, s), 1.87 (3.2H, s), 1.85 (1.5H, s), 1.74 (1.3H, s) ppm. MALDI-TOF (pos) *m*/*z* 39,700.

**8c**: ^1^H-NMR (500 MHz *d*_6_-DMSO) δ 7.95 (1H, bs, amide NH's), 7.79 (0.9H, d, *J* = 9.0 Hz, N*H*'Ac), 7.74 (0.9H, bs, amide NH's), 7.46 (2.1H, bs, CH_2_N*H*C(S)N*H*CH_2_), 5.21 (0.7H, d, *J* = 3.2 Hz, H4), 5.17 (0.7H, s, H4'), 5.11 (0.7H, d, *J* = 10.3 Hz, H3), 4.92 (0.7H, d, *J* = 10.3 Hz, H3'), 4.89 (0.7H, t, *J* = 9.7 Hz, H2), 4.69 (0.6H, d, *J* = 7.6 Hz, H1), 4.51 (0.7H, d, *J* = 8.4 Hz, H1'), 4.12 (0.8H, m), 4.01 (3.9H, m), 3.83 (0.7H, app q, *J* = 8.4, 9.0, 10.3 Hz, H2'), 3.76 (1.9H, m), 3.27–3.59 (13.6H, m), 3.13 (2.9H, bs), 3.04 (2.4H, bs), 2.61 (5.3H, m), 2.39 (2.6H, m), 2.16 (4.6H, bs), 2.08 (5H, s), 2.00 (4.8H, s), 1.96 (1.9H, s), 1.87 (2.4H, s) 1.74 (2.1H, s) ppm. MALDI-TOF (pos) *m*/*z* 40,200.

**8d**: ^1^H-NMR (500 MHz *d*_6_-DMSO) δ 7.95 (1H, bs, amide NH's), 7.80 (1.0H, d, *J* = 9.0 Hz, N*H*'Ac), 7.79 (0.8H, bs, amide NH's), 7.46 (2.0H, m, CH_2_N*H*C(S)N*H*CH_2_), 5.21 (0.5H, s, H4), 5.17 (0.9H, s, H4'), 5.11 (0.4H, d, 10.3 Hz, H3), 4.92 (1H, d, *J* = 10.3 Hz, H3'), 4.89 (0.4H, t, *J* = 9.7 Hz, H2), 4.69 (0.4H, d, *J* = 7.6 Hz, H1), 4.51 (0.9H, d, *J* = 8.4 Hz, H1'), 4.12 (0.5H, m), 4.01 (4.1H, m), 3.83 (1.1H, app q, *J* = 8.4, 9.0, 10.3 Hz, H2'), 3.76 (2.0H, m), 3.27–3.59 (13.1H, m), 3.13 (2.7H, bs), 3.04 (2.0H, bs), 2.61 (4.2H, m), 2.39 (2.6H, m), 2.16 (3.8H, bs), 2.07 (1.7H, s), 2.06 (3.9H, s), 1.98 (1H, s), 1.96 (5.3H, s), 1.87 (1.4H, s), 1.85 (2.9H, s), 1.74 (2.8H, s) ppm. MALDI-TOF (pos) *m*/*z* 39,700.

**8e**: ^1^H-NMR (500 MHz *d*_6_-DMSO) δ 7.95 (1H, bs, amide NH's), 7.79 (1.3H, d, *J* = 9.0 Hz, N*H*'Ac), 7.74 (0.8H, bs, amide NH's), 7.46 (2.1H, m, CH_2_N*H*C(S)N*H*CH_2_), 5.21 (0.3H, d, *J* = 3.2 Hz, H4), 5.17 (1.2H, s, H4'), 5.11 (0.3H, d, *J* = 10.3 Hz, H3), 4.92 (1.2H, d, *J* = 10.3 Hz, H3'), 4.89 (0.3H, t, *J* = 9.7 Hz, H2), 4.69 (0.2H, d, *J* = 7.6 Hz, H1), 4.51 (1.2H, d, *J* = 8.4 Hz, H1'), 4.12 (0.3H, m), 4.01 (3.6H, m), 3.83 (1.3H, app q, *J* = 8.4, 9.0, 10.3 Hz, H2'), 3.76 (2.2H, m), 3.27–3.59 (8.3H, m), 3.13 (3.0H, bs), 3.04 (2.1H, bs), 2.61 (5.0H, m), 2.39 (2.7H, m), 2.16 (4.4H, bs), 2.07 (1.1H, s), 2.06 (3.4H, s), 1.95 (5.3H, s), 1.87 (1H, s), 1.85 (3.9H, s), 1.74 (3.7H, s) ppm. MALDI-TOF (pos) *m*/*z* 40,500.

**8f**: ^1^H-NMR (500 MHz *d*_6_-DMSO) δ 7.96 (1H, bs, amide NH's), 7.81 (1.2H, d, *J* = 9.0 Hz, N*H*'Ac), 7.74 (0.8H, bs, amide NH's), 7.46 (2.0H, m, CH_2_N*H*C(S)N*H*CH_2_), 5.17 (1.3H, s, H4'), 4.92 (1.3H, d, *J* = 10.3 Hz, H3'), 4.51 (1.4H, d, *J* = 8.4 Hz, H1'), 4.01 (4.9H, m), 3.83 (1.6H, app q, *J* = 8.4, 9.0, 10.3 Hz, H2'), 3.27–3.59 (17H, m), 3.13 (2.8H, bs), 3.04 (2.1H, bs), 2.61 (4H, m), 2.39 (2H, m), 2.16 (3.9H, bs), 2.06 (4.7H, s), 1.95 (5H, s), 1.85 (4.5H, s), 1.74 (4.4H, s) ppm. MALDI-TOF (pos) *m*/*z* 39,300.

**9a**: ^1^H-NMR (500 MHz *d*_6_-DMSO) δ 7.95 (1H, bs, amide NH's), 7.74–7.79 (1.3H, m, amide NH's, N*H*'Ac), 7.45 (1.9H, bs, CH_2_N*H*C(S)N*H*CH_2_), 5.21 (1H, s, H4), 5.17 (0.2H, s, H4'), 5.11 (1H, d, 10.3 Hz, H3), 4.89 (1H, t, *J* = 9.7 Hz, H2), 4.69 (1H, d, *J* = 7.6 Hz, H1), 4.51 (0.2H, d, *J* = 8.4 Hz, H1'), 4.12 (1H, m), 4.01 (2.6H, m), 3.83 (0.2H, app q, *J* = 8.4, 9.0, 10.3 Hz, H2'), 3.76 (1.5H, m), 3.27–3.59 (11.4H, m), 3.13 (2.9H, bs), 3.04 (2.3H, bs), 2.61 (4.8H, m), 2.16 (4.1H, bs), 2.08 (4.2H, s), 1.98 (7.6H, s), 1.87 (3.7H, s) 1.72 (0.8H, s) ppm. MALDI-TOF (pos) *m*/*z* 119,500.

**9b**: ^1^H-NMR (500 MHz *d*_6_-DMSO) δ 7.96 (1H, bs, amide NH's), 7.79 (0.6H, d, *J* = 9.0 Hz, N*H*'Ac), 7.74 (0.9H, bs, amide NH's), 7.46 (1.9H, bs, CH_2_N*H*C(S)N*H*CH_2_), 5.21 (2.1H, d, *J* = 3.3 Hz, H4), 5.17 (0.4H, s, H4'), 5.11 (2.1H, dd, *J* = 3.2, 10.3 Hz, H3), 4.92 (0.3H, d, *J* = 10.3 Hz, H3'), 4.89 (2.1H, t, *J* = 9.7 Hz, H2), 4.69 (1.9H, d, *J* = 7.6 Hz, H1), 4.51 (0.2H, d, *J* = 8.4 Hz, H1'), 4.12 (2.1H, m), 4.01 (5.4H, m), 3.83 (0.3H, app q, *J* = 8.4, 9.0, 10.3 Hz, H2'), 3.76 (5.4H, m), 3.27–3.59 (17.5H, m), 3.13 (3.2H, bs), 3.04 (2.3H, bs), 2.61 (4.8H, m), 2.39 (2.8H, m), 2.16 (4.4H, bs), 2.08 (7.8H, s), 2.06 (4.6H, s), 1.96 (5.5H, s), 1.85 (1.6H, s), 1.74 (1.4H, s) ppm. MALDI-TOF (pos) *m*/*z* 119,000.

**9c**: ^1^H-NMR (500 MHz *d*_6_-DMSO) δ 7.95 (1H, bs, amide NH's), 7.81 (0.7H, d, *J* = 9.0 (H2') Hz, N*H*'Ac), 7.74 (0.9H, bs, amide NH's), 7.46 (2H, bs, CH_2_N*H*C(S)N*H*CH_2_), 5.21 (0.7H, d, *J* = 3.2 Hz, H4), 5.17 (0.6H, s, H4'), 5.11 (0.7H, d, *J* = 10.3 Hz, H3), 4.92 (0.6H, d, *J* = 10.3 Hz, H3'), 4.89 (0.7H, t, *J* = 9.7 Hz, H2), 4.69 (0.6H, d, *J* = 7.6 Hz, H1), 4.51 (0.5H, d, *J* = 8.4 Hz, H1'), 4.12 (0.8H, m), 4.01 (3.2H, m), 3.83 (0.7H, app q, *J* = 8.4, 9.0, 10.3 Hz, H2'), 3.76 (1.7H, m), 3.27–3.59 (12H, m), 3.13 (3H, bs), 3.04 (2.1H, bs), 2.61 (5.4H, m), 2.39 (2H, m), 2.16 (4H, bs), 2.08 (3.7H, s), 2.00 (5.5H, s), 1.96 (1.7H, s), 1.87 (1.6H, s), 1.74 (1.4H, s) ppm. MALDI-TOF (pos) *m*/*z* 119,000.

**9d**: ^1^H-NMR (500 MHz *d*_6_-DMSO) δ 7.96 (1H, bs, amide NH's), 7.79 (0.8H, d, *J* = 9.0 Hz, N*H*'Ac), 7.74 (0.7H, bs, amide NH's), 7.46 (1.8H, m, CH_2_N*H*C(S)N*H*CH_2_), 5.21 (0.5H, s, H4), 5.17 (0.6H, s, H4'), 5.11 (0.5H, d, *J* = 10.3 Hz, H3), 4.92 (0.6H, d, *J* = 10.3 Hz, H3'), 4.89 (0.5H, t, *J* = 9.7 Hz, H2), 4.69 (0.4H, d, *J* = 7.6 Hz, H1), 4.51 (0.6H, d, *J* = 8.4 Hz, H1'), 4.12 (0.6H, m), 4.01 (3.1H, m), 3.83 (0.8H, app q, *J* = 8.4, 9.0, 10.3 Hz, H2'), 3.76 (1.5H, m), 3.27–3.59 (10H, m), 3.13 (2.8H, bs), 3.04 (1.7H, bs), 2.61 (4H, m), 2.39 (2.2H, m), 2.16 (3.5H, bs), 2.07 (3.5H, s), 1.98 (0.7H, s), 1.96 (4.4H, s), 1.87 (1.2H, s), 1.85 (2H, s), 1.74 (1.9H, s) ppm. MALDI-TOF (pos) *m*/*z* 121,500.

**9e**: ^1^H-NMR (500 MHz *d*_6_-DMSO) δ 7.95 (1H, bs, amide NH's), 7.79 (0.8H, d, *J* = 9.0 Hz, N*H*'Ac), 7.74 (0.7H, bs, amide NH's), 7.46 (1.6H, bs, CH_2_N*H*C(S)N*H*CH_2_), 5.21 (0.2H, d, *J* = 3.2 Hz, H4), 5.17 (0.7H, s, H4'), 5.11 (0.3H, m, Hz, H3), 4.92 (0.7H, d, *J* = 10.3 Hz, H3'), 4.89 (0.2H, t, *J* = 9.7 Hz, H2), 4.69 (0.2H, d, *J* = 7.6 Hz, H1), 4.51 (0.7H, d, *J* = 8.4 Hz, H1'), 4.12 (0.3H, m), 4.01 (2.9H, m), 3.83 (0.9H, app q, *J* = 8.4, 9.0, 10.3 Hz, H2'), 3.7 (1.2H, m), 3.27–3.59 (8.6H, m), 3.04–3.13 (2.8H, m), 2.61 (3.2H, m), 2.39 (1.4H, m), 2.16 (2.9H, bs), 2.07 (3.2H, s), 1.95 (3.9H, s), 1.85 (2.9H, s), 1.74 (2.2H, s) ppm. MALDI-TOF (pos) *m*/*z* 121,500.

**9f**: ^1^H-NMR (500 MHz *d*_6_-DMSO) δ 7.96 (1H, bs, amide NH's), 7.79 (1.1H, d, *J* = 9.0 Hz, N*H*'Ac), 7.74 (0.8H, bs, amide NH's), 7.46 (2.8H, m, CH_2_N*H*C(S)N*H*CH_2_), 5.17 (1.1H, s, H4'), 4.92 (1.1H, d, *J* = 10.3 Hz, H3'), 4.51 (1.2H, d, *J* = 8.4 Hz, H1'), 4.01 (4.2H, m), 3.83 (1.2H, app q, *J* = 8.4, 9.0, 10.3 Hz, H2'), 3.27–3.59 (12.7H, m), 3.04–3.13 (4.2H, m), 2.61 (3.9H, m), 2.16 (3.1H, bs), 2.06 (4.4H, s), 1.95 (4.1H, s), 1.85 (3.7H, s), 1.74 (3.8H, s) ppm. MALDI-TOF (pos) *m*/*z* 125,000.

##### Deacetylated

**7a**: ^1^H-NMR (500 MHz *d*_6_-DMSO) δ 8.00 (bs, 1H), 7.81 (bs, 1H), 7.67 (d, *J* = 8.6 Hz, 0.2H), 7.50 (bs, 2.1H), 4.95 (bs, 0.7H), 4.60–4.70 (m, 1.5H), 4.43 (bs, 0.8H), 4.27 (d, *J* = 8.3 Hz, 0.2H), 4.11 (d, *J* = 5.2 Hz, 0.8H), 3.84 (m, 1.0H), 3.38–3.65 (m, 27H), 3.17 (bs, 2.8H), 3.08 (bs, 2.6H), 2.66 (bs, 4.0H), 2.42 (bs, 2.3H), 2.20 (bs, 4.3H), 1.80 (m, 0.9H) ppm. MALDI-TOF (pos) *m*/*z* 15,000.

**7b**: ^1^H-NMR (500 MHz *d*_6_-DMSO) δ 8.00 (bs, 1H), 7.80 (bs, 0.9H), 7.67 (d, *J* = 8.6 Hz, 0.3H), 7.50 (m, 1.5H), 4.95 (bs, 0.6H), 4.50–4.75 (m, 2.1H), 4.43 (bs, 0.7H), 4.27 (d, *J* = 8.3 Hz, 0.4H), 4.11 (d, *J* = 5.2Hz, 0.7H), 3.84 (m, 0.9H), 3.38–3.65 (m, 24H), 3.17 (bs, 2.3H), 3.08 (bs, 1.9H), 2.66 (bs, 3.4H), 2.42 (bs, 1.8H), 2.20 (bs, 3.7H), 1.80 (m, 1.0H) ppm. MALDI-TOF (pos) *m*/*z* 15,100.

**7c**: ^1^H-NMR (500 MHz *d*_6_-DMSO) δ 8.01 (bs, 1H), 7.83 (bs, 0.9H), 7.67 (d, *J* = 8.6 Hz, 0.5H), 7.59 (bs, 0.5H), 7.50 (bs, 1.2H), 4.95 (bs, 0.5H), 4.60–4.70 (m, 2.2H), 4.43 (bs, 0.7H), 4.27 (d, *J* = 8.3 Hz, 0.7H), 4.11 (d, *J* = 5.2 Hz, 0.6H), 3.84 (m, 1.0H), 3.38–3.65 (m, 30H), 3.17 (bs, 3.1H), 3.08 (bs, 2.1H), 2.66 (bs, 4.0H), 2.42 (bs, 1.8H), 2.20 (bs, 3.9H), 1.80 (m, 1.6H) ppm. MALDI-TOF (pos) *m*/*z* 7,900.

**7d**: ^1^H-NMR (500 MHz *d*_6_-DMSO) δ 8.02 (bs, 1H), 7.83 (bs, 0.8H), 7.67 (d, *J* = 8.6 Hz, 0.7H), 7.59 (bs, 0.6H), 7.50 (m, 1.1H), 4.95 (bs, 0.4H), 4.60–4.70 (m, 2.4H), 4.43 (bs, 0.5H), 4.27 (d, *J* = 8.3 Hz, 0.9H), 4.11 (d, *J* = 5.2 Hz, 0.5H), 3.38–3.65 (m, 30H), 3.17 (bs, 2.9H), 3.08 (bs, 2.0H), 2.66 (bs, 3.9H), 2.42 (bs, 1.9H), 2.20 (bs, 3.9H), 1.80 (m, 2.0H) ppm. MALDI-TOF (pos) *m*/*z* 15,700.

**7e**: ^1^H-NMR (500 MHz *d*_6_-DMSO) δ 8.01 (bs, 1H), 7.82 (bs, 0.9H), 7.67 (d, *J* = 8.6 Hz, 0.9H), 7.60 (bs, 0.8H), 7.50 (bs, 0.7H), 4.95 (bs, 0.2H), 4.60–4.70 (m, 2.6H), 4.43 (bs, 0.2H), 4.27 (d, *J* = 8.3 Hz, 1.0H), 4.11 (d, *J* = 5.2 Hz, 0.3H), 3.38–3.65 (m, 23H), 3.17 (bs, 2.6H), 3.08 (bs, 2.61), 2.66 (bs, 3.9H), 2.42 (bs, 1.7H), 2.20 (bs, 3.7H), 1.80 (m, 2.6H) ppm. MALDI-TOF (pos) *m*/*z* 15,800.

**7f**: ^1^H-NMR (500 MHz *d*_6_-DMSO) δ 8.01 (bs, 1H), 7.82 (bs, 0.9H), 7.68 (d, *J* = 8.6 Hz, 1.0H), 7.59 (bs, 0.9H), 7.50 (m, 1.0H), 4.60–4.70 (m, 2.8H), 4.27 (d, *J* = 8.3 Hz, 1.1H), 3.77 (m, 3.3H), 3.38–3.65 (m, 30H), 3.17 (bs, 3.3H), 3.10 (bs, 1.9H), 2.66 (bs, 4.1H), 2.42 (bs, 1.9H), 2.20 (bs, 3.8H), 1.80 (m, 3.0H) ppm. MALDI-TOF (pos) *m*/*z* 16,400.

**8a**: ^1^H-NMR (500 MHz *d*_6_-DMSO) δ 8.01 (bs, 1H), 7.81 (bs, 1H), 7.67 (m, 0.2H), 7.50 (bs, 1.8H), 4.95 (bs, 0.6H), 4.60–4.70 (m, 2.0H), 4.43 (bs, 0.9H), 4.27 (m, 0.2H), 4.11 (m, 0.8H), 3.84 (m, 1.0H), 3.38–3.65 (m, 30H), 3.17 (bs, 2.5H), 3.08 (bs, 2.6H), 2.66 (bs, 3.9H), 2.42 (bs, 2.1H), 2.20 (bs, 4.1H), 1.80 (m, 0.7H) ppm. 31,200.

**8b**: ^1^H-NMR (500 MHz *d*_6_-DMSO) δ 8.03 (bs, 1H), 7.85 (bs, 0.9H), 7.73 (d, *J* = 8.6 Hz, 0.4H), 7.50 (m, 1.6H), 4.95 (bs, 0.4H), 4.50–4.90 (m, 2.3H), 4.52 (bs, 0.6H), 4.27 (d, *J* = 8.3 Hz, 0.5H), 4.12 (s, 0.9H), 3.38–3.70 (m, 30H), 3.17 (bs, 2.7H), 3.08 (bs, 2.1H), 2.66 (bs, 3.8H), 2.42 (bs, 1.8H), 2.20 (bs, 3.9H), 1.80 (m, 1.2H) ppm. MALDI-TOF (pos) *m*/*z* 31,700.

**8c**: ^1^H-NMR (500 MHz *d*_6_-DMSO) δ 8.02 (bs, 1H), 7.83 (bs, 0.9H), 7.71 (m, 0.5H), 7.58 (bs, 0.6H), 7.50 (bs, 1.0H), 4.95 (bs, 0.3H), 4.60–4.70 (m, 1.7H), 4.58 (bs, 0.5H), 4.48 (bs, 0.5H), 4.27 (d, *J* = 8.3 Hz, 0.5H), 4.11 (m, 0.4H), 3.84 (m, 1.6H), 3.38–3.65 (m, 30H), 3.17 (bs, 2.4H), 3.08 (bs, 2.1H), 2.66 (bs, 3.8H), 2.42 (bs, 1.7H), 2.20 (bs, 4.0H), 1.80 (m, 1.8H) ppm. MALDI-TOF (pos) *m*/*z* 33,000.

**8d**: ^1^H-NMR (500 MHz *d*_6_-DMSO) δ 8.02 (bs, 1H), 7.83 (bs, 0.8H), 7.67 (d, *J* = 8.6 Hz, 0.8H), 7.58 (bs, 0.7H), 7.43 (m, 0.9H), 4.95 (bs, 0.1H), 4.69 (bs, 1.7H),4.58 (bs, 0.8H), 4.43 (bs, 0.2H), 4.27 (d, *J* = 8.3 Hz, 0.8H), 4.11 (m, 0.1H), 3.38–3.65 (m, 30H), 3.17 (bs, 2.5H), 3.08 (bs, 1.8H), 2.66 (bs, 4.1H), 2.42 (bs, 1.8H), 2.21 (bs, 3.8H), 1.80 (m, 2.2H) ppm. MALDI-TOF (pos) *m*/*z* 34,300.

**8e**: ^1^H-NMR (500 MHz *d*_6_-DMSO) δ 8.02 (bs, 1H), 7.83 (bs, 0.8H), 7.67 (d, *J* = 8.6 Hz, 0.9H), 7.58 (bs, 0.8H), 7.44 (bs, 0.8H), 4.68 (m, 1.5H), 4.58 (1.0H), 4.27 (d, *J* = 8.3 Hz, 0.9H), 3.38–3.65 (m, 23H), 3.17 (bs, 2.4H), 3.08 (bs, 1.9H), 2.66 (bs, 3.8H), 2.42 (bs, 1.6H), 2.20 (bs, 3.8H), 1.80 (m, 2.8H) ppm. MALDI-TOF (pos) *m*/*z* 34,300.

**8f**: ^1^H-NMR (500 MHz *d*_6_-DMSO) δ 8.02 (bs, 1H), 7.83 (bs, 0.8H), 7.69 (d, *J* = 8.6 Hz, 0.8H), 7.58 (bs, 0.8H), 7.43 (m, 0.9H), 4.67 (m, 1.6H), 4.58 (bs, 0.8H), 4.27 (d, *J* = 8.3 Hz, 0.8H), 3.38–3.65 (m, 30H), 3.10–3.17 (m, 4.6H), 2.66 (bs, 3.7H), 2.42 (bs, 1.6H), 2.20 (bs, 3.6H), 1.80 (m, 2.4H) ppm. MALDI-TOF (pos) *m*/*z* 33,300.

**9a**: ^1^H-NMR (500 MHz *d*_6_-DMSO) δ 8.01 (bs, 1H), 7.83 (bs, 0.7H), 7.67 (m, 0.2H), 7.48 (bs, 1.5H), 5.04 (bs, 0.7H), 4.60–4.70 (m, 1.7H), 4.43 (bs, 0.8H), 4.28 (m, 0.2H), 4.11 (s, 1.0H), 3.84 (m, 2.0H), 3.38–3.65 (m, 30H), 3.17 (bs, 2.1H), 3.08 (bs, 1.7H), 2.65 (bs, 3.2H), 2.42 (bs, 1.4H), 2.20 (bs, 3.5H), 1.80 (m, 0.6H) ppm. MALDI-TOF (pos) *m*/*z* 100,000.

**9b**: ^1^H-NMR (500 MHz *d*_6_-DMSO) δ 8.03 (bs, 1H), 7.85 (bs, 0.7H), 7.73 (s, 0.3H), 7.47 (m, 1.4H), 5.07 (bs, 0.5H), 4.68–4.90 (m, 1.7H), 4.63 (bs, 0.4H), 4.54 (bs, 0.6H), 4.27 (s, 0.4H), 4.12 (s, 0.9H), 3.38–3.70 (m, 30H), 3.17 (bs, 2.2H), 3.08 (bs, 1.6H), 2.66 (bs, 2.8H), 2.20 (bs, 3.4H), 1.80 (m, 1.2H) ppm. MALDI-TOF (pos) *m*/*z* 101,000.

**9c**: ^1^H-NMR (500 MHz *d*_6_-DMSO) δ 8.02 (bs, 1H), 7.84 (bs, 0.7H), 7.72 (s, 0.5H), 7.58 (bs, 0.5H), 7.44 (bs, 0.9H), 5.04 (bs, 0.3H), 4.72 (m, 1.7H), 4.61 (bs, 0.8H), 4.28 (s, 0.6H), 4.13 (m, 0.4H), 3.38–3.80 (m, 30H), 3.10–3.17 (m, 3.5H), 2.66 (bs, 3.0H), 2.42 (bs, 1.0H), 2.20 (bs, 3.4H), 1.80 (m, 1.5H) ppm. MALDI-TOF (pos) *m*/*z* 101,500.

**9d**: ^1^H-NMR (500 MHz *d*_6_-DMSO) δ 8.02 (bs, 1H), 7.84 (bs, 0.8H), 7.72 (s, 0.4H), 7.57 (bs, 0.5H), 7.48 (m, 0.9H), 5.05 (bs, 0.4H), 4.69 (bs, 1.6H), 4.58 (bs, 0.5H), 4.43 (bs, 0.2H), 4.27 (s, 0.5H), 4.13 (m, 0.5H), 3.38–3.65 (m, 30H), 3.10–3.17 (bs, 3.6H), 2.66 (bs, 3.0H), 2.42 (bs, 1.3H), 2.21 (bs, 3.3H), 1.82 (m, 1.4H) ppm. MALDI-TOF (pos) *m*/*z* 102,000.

**9e**: ^1^H-NMR (500 MHz *d*_6_-DMSO) δ 8.02 (bs, 1H), 7.82 (bs, 0.7H), 7.67 (s, 0.7H), 7.58 (bs, 0.7H), 7.44 (bs, 0.8H), 5.05 (bs, 0.2H), 4.68 (m, 1.7H), 4.60 (0.7H), 4.28 (s, 0.7H), 3.38–3.65 (m, 23H), 3.10–3.17 (m, 3.6H), 2.66 (bs, 2.9H), 2.42 (bs, 0.8H), 2.21 (bs, 3.2H), 1.80 (m, 1.8H) ppm. MALDI-TOF (pos) *m*/*z* 106,500.

**9f**: ^1^H-NMR (500 MHz *d*_6_-DMSO) δ 8.03 (bs, 1H), 7.84 (bs, 0.7H), 7.72 (s, 0.8H), 7.58 (bs, 0.7H), 7.43 (m, 0.9H), 4.73 (m, 1.6H), 4.62 (bs, 0.9H), 4.27 (s, 0.8H), 3.38–3.65 (m, 30H), 3.10–3.17 (m, 3.8H), 2.66 (bs, 3.0H), 2.42 (bs, 1.2H), 2.21 (bs, 3.5H), 1.80 (m, 2.2H) ppm. MALDI-TOF (pos) *m*/*z* 107,500.

### 3.6. ELISA Methods

#### 3.6.1. Preparation of Glycodendrimer Adsorbed 96 well Plates

Glycodendrimer was dissolved into PBS (pH 7.4, 15 mmol NaCl). The stock solution was commonly prepared at 5 mg/mL, but the concentration of the stock solution was reduced if there were problems with solubility. To each well of a Nunc MaxiSorp 96 well plate (Thermo Scientific, Waltham, MA, USA), 50 µL of a 0.025 mg/mL solution was added. The well plate was covered and stored for 24 h at 4 °C. The solvent was removed from the well plate and 250 μL of 3% BSA solution in PBS (pH 7.4, 15 mmol NaCl) was added to each well plate to block non-specific interactions. The plate was covered and let stand for 2 h at RT. After 2 h, the plate was emptied, washed once with PBS (pH 7.4, 15 mmol NaCl), and dried. Dried plates were either used immediately or covered and stored at 4 °C for later use.

#### 3.6.2. ELISA to Study Galectin-3 Binding Interactions with Glycodendrimers

In a PPI plate, 60 μL of 0.5% BSA in PBS (pH 7.4) was added to each well, except A1, C1, E1 and G1. To A1, C1, E1, and G1, 60 μL of 100 mg/mL lactose solution were added. To A2, C2, E2, and G2, 60 μL of the 100 mg/mL lactose solution were added. To generate 24 sequential lactose concentrations, serial dilutions were performed, starting with wells A2, D2, and G2, so that 60 µL remained in each well. From each well, 50 μL were transferred to the corresponding well on the glycodendrimer coated prepared plate (preparation of the glycodendrimer coated plate is described above in 4.1.1.). Galectin-3 was added (50 μL of 10 μg/mL solution, concentration determined using a BCA assay [88]), the plate was covered and placed on an agitator/shaker for 45 min.

After 45 min, the plate was removed from the shaker and the contents were emptied. Each well was washed 2× with PBS-T (pH 7.4) and 1× with PBS (pH 7.4). Biotinylated anti-galectin-3 was added (50 μL of a 1:100 dilution of 1 mL stock from R and D Systems, Inc., Minneapolis, MN, USA), the plate was covered and placed back on the shaker for 45 min. After 45 min, the plate was removed from the shaker and the wells were emptied and each well was washed 2× with PBS-T (pH 7.4) and 1× with PBS (pH 7.4). Horseradish peroxidase streptavidin conjugate was added (100 μL of solution that was a 1:200 dilution from the stock obtained from BD Biosciences, Seattle, WA, USA), and the plate was covered and placed on the shaker for 45 min. The plate was removed from the shaker, the wells were emptied and each well was washed 2× with PBS-T (pH 7.4) and 1× with PBS (pH 7.4).

TMB (tetramethylbenzidene): peroxide solution (100 μL of a 1:1 mix of solutions from kit purchased from BD biosciences) was added and the color change was observed. 100 μL of 1 M phosphoric acid was added to stop the reaction (This can be monitored at 620 nm on a plate reader). Absorbances were read at 450 nm for each well plate, with the reference at 620 nm.

#### 3.6.3. ELISA to Study Galectin-1 Binding Interactions with Glycodendrimers

To a processed polystyrene (PPI) plate, 60 µL of 0.5% BSA in PBS (pH 7.4, 15 mM NaCl) were added to all wells, except A1, D1 and G1. Wells A1, D1, and G1 were filled with 60 µL of a 100 mg/mL lactose solution. To wells A2, D2, and G2, 60 µL of a 100 mg/mL lactose solution were added. To generate 24 sequential lactose concentrations, serial dilutions were performed, starting with wells A2, D2, and G2, so that 60 µL remained in each well. From each well on the PPI plate, 50 µL were transferred to the corresponding well on the glycodendrimer coated plate (preparation of the glycodendrimer coated plate is described above in 3.6.1.). To all wells, biotinylated galectin-1 (50 µL of a 5.0 µg/mL solution) was added (biotinylation of galectin-1 is described in Section 3.4). The plate was covered and placed on a shaker/agitator for 45 min.

After 45 min, the plate was emptied and washed, twice with PBS-T (pH 7.4, 15 mM NaCl) and once with PBS (pH 7.4, 15 mM NaCl). 100 µL of streptavidin-horseradish peroxidase (SAv-HRP) (1:1000 dilution of the stock purchased from BD Biosciences) were added to each well. The plate was covered and placed on a shaker/agitator for 45 min.

After 45 min, the plate was emptied and washed, twice with PBS-T (pH 7.4, 15 mM NaCl) and once with PBS (pH 7.4, 15 mM NaCl). 100 µL of TMB/H_2_O_2_ (mixed in a 1:1 ratio from a kit purchased from BD Biosciences) were added to each well. The plate was covered and incubated for 10 min. A blue color change was observed. After 10 min, 100 µL of H_3_PO_4_ was added to each well and a yellow color change was observed. Absorbances were measured at 450 nm, with a reference at 620 nm.

#### 3.6.4. Analysis of ELISA Binding Curves

Data collected from the ELISAs were analysed with GraphPad Prism software (Version 5, GraphPad Software, Inc. La Jolla, CA, USA), which generated logarithmic binding curves as a function of the log of the concentration of lactose (mM). 

### 3.7. X-ray Photoelectron Spectroscopy (XPS)

X-ray photoelectron spectroscopy was used to analyze the amount of glycodendrimer that adsorbed to the Nunc MaxiSorp 96 well plate (Thermo Scientific, Waltham, MA, USA) surface. Experiments were performed with compounds **4a**, **5a**, **6a**, **4f**, **5f**, **6f**. To monitor the amount of glycodendrimer adsorbed to the plate, the amount of nitrogen was used as a quantitative measurement. Since the Nunc MaxiSorp plates have oxygen in the surface coating to facilitate surface adsorption, a background signal in the oxygen spectrum is always present. The entire nitrogen spectrum, however, is due solely to the glycodendrimer. The XPS experiments were performed with higher concentrations of glycodendrimer than the ELISA to increase the adsorbed glycodendrimer to the plate surface, hence increasing the signal in the nitrogen spectrum. Experiments with very high amounts of dendrimer in the solution during the adsorption revealed little or no difference between 5 μmol and 50 μmol solutions, with 5 μmol being the optimal and preferred concentration for XPS experiments. The ELISA assay adsorption concentration was 250 nmol; at this concentration the same XPS trends were observed although the nitrogen signal was decreased and the sulfur signal was too small to be observable.

Carbohydrate functionalized dendrimers were dissolved in a PBS solution at 1 mg/mL concentration, which required 24 h of stirring. These solutions were diluted with PBS buffer to a concentration of 5 μM. 50 μL of this solution was added to a well of a Nunc MaxiSorp 96 well plate, covered and stored at 5 °C for 20 h. The well was washed with PBS twice and washed with nanopure water twice to remove any phosphate buffer that might interfere with the analysis. The bottom of the well plate was removed with scissors and was used for XPS analysis. The analysis was conducted on a Physical Electronics 5600ci XPS system equipped with monochromatized Al KR X-rays. The analysis area of the sample was 0.8 mm in diameter. Electron emissions were collected at 45° to the normal of the surface, and the spherical-sector-analyser pass energy was selected as 11.75 eV for high-resolution scanning and as 46.95 eV for a survey to achieve optimum energy resolution and count rate. The data acquisition and data analysis were performed using RBD AugerScan 2 software (Version 2, RBD Instruments, Bend, OR, USA).

## 4. Conclusions

In summary, generation three, four, and six PAMAM dendrimers bearing heterogeneous mixtures of β-galactoside, β-GalNAc, and β-lactoside were synthesized and characterized. This small library of glycodendrimers was used to assess multivalent effects in a complex carbohydrate/galectin-3 interaction. A modified ELISA based experiment was developed to further the understanding of how galectin-3 binds to carbohydrates. This assay revealed that galectin-3 interacts with glycodendrimers in a markedly different way than it interacts with monomeric sugars. The multivalent binding constants that were determined using the modified ELISA assay for the dendrimers with galectin-3 are all very similar, even with sugar epitopes that have binding constants that differ by almost two orders of magnitude. However, the modified ELISA revealed that galectin-3 recruitment was directly dependent on the ratio of low to high affinity ligands on the dendrimers. To validate the utility of the modified ELISA for study of multivalent binding by galectins, lactose functionalized dendrimers of three generations of dendrimers were studied in the same modified ELISA format with galectin-1. Galectin-1 recruitment was shown to be dependent on dendrimer generation/valency, while IC_50_ values were determined to be consistent regardless of the generation of dendrimer that was used. The results with galectin-1 and galectin-3 are highly complementary and indicate the prevalence of a multivalent binding interaction between glycodendrimers and these galectins. 

The observations detailed here indicate that multivalent architectures are likely to be critical for improving the understanding of the behaviour of the galectins in biological recognition events. These results suggest that the binding affinity may play less of a role in galectin mediated processes than clustering and aggregation mechanisms. In general, an in-depth understanding of the aggregation that occurs in complex systems requires studies using many different assays in many different formats. Because of the complexity and the high prevalence of biologically relevant multivalent interactions, relatively straightforward ways of describing, defining, and characterizing multivalent protein-carbohydrate interactions such as this modified ELISA are essential.

## Supplementary Materials

Supplementary materials can be accessed at: http://www.mdpi.com/1420-3049/20/04/7059/s1.
