# Supplementary material for: Glycodendrimers and Modified ELISAs: Tools to Elucidate Multivalent Interactions of Galectins 1 and 3
Source: Molecules. 2015 Apr 20;20(4):7059–96. doi: 10.3390/molecules20047059 (PMC4513649; doi:10.3390/molecules20047059)
Supplement: Supplementary file 1 [file molecules-20-07059-s001.pdf]

Supplementary Materials

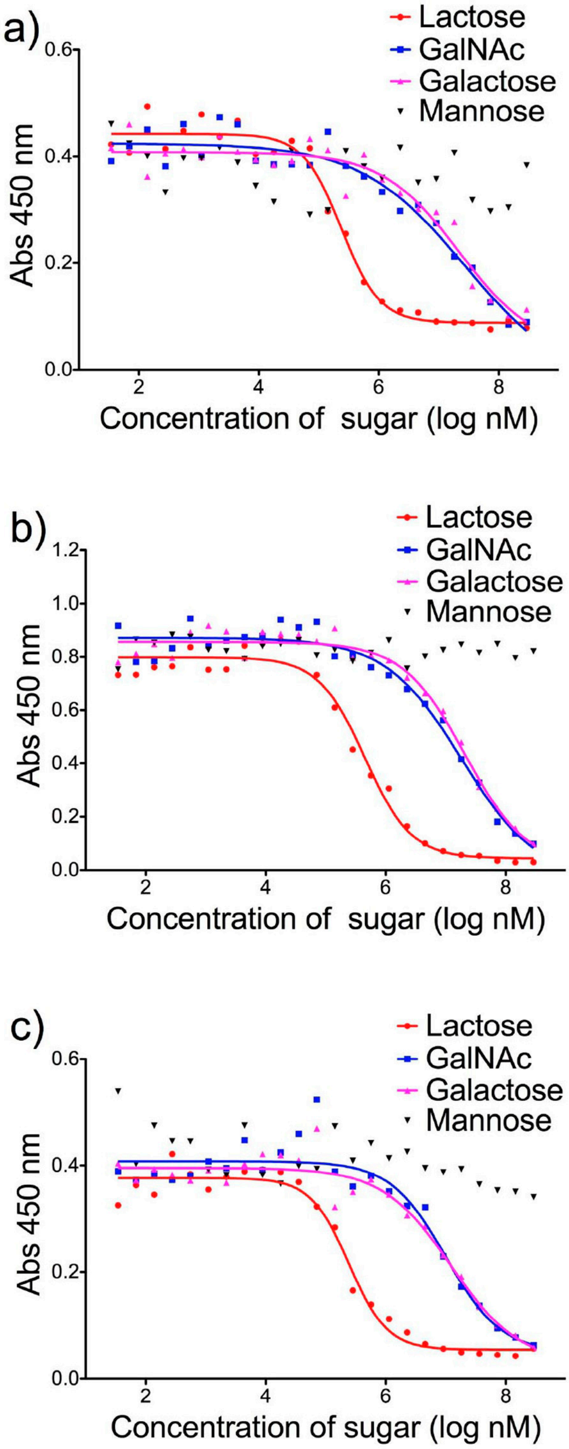

Figure S1. ELISA curves with different inhibitors with (**a**) **4a**; (**b**) **5a** and (**c**) **6a**.

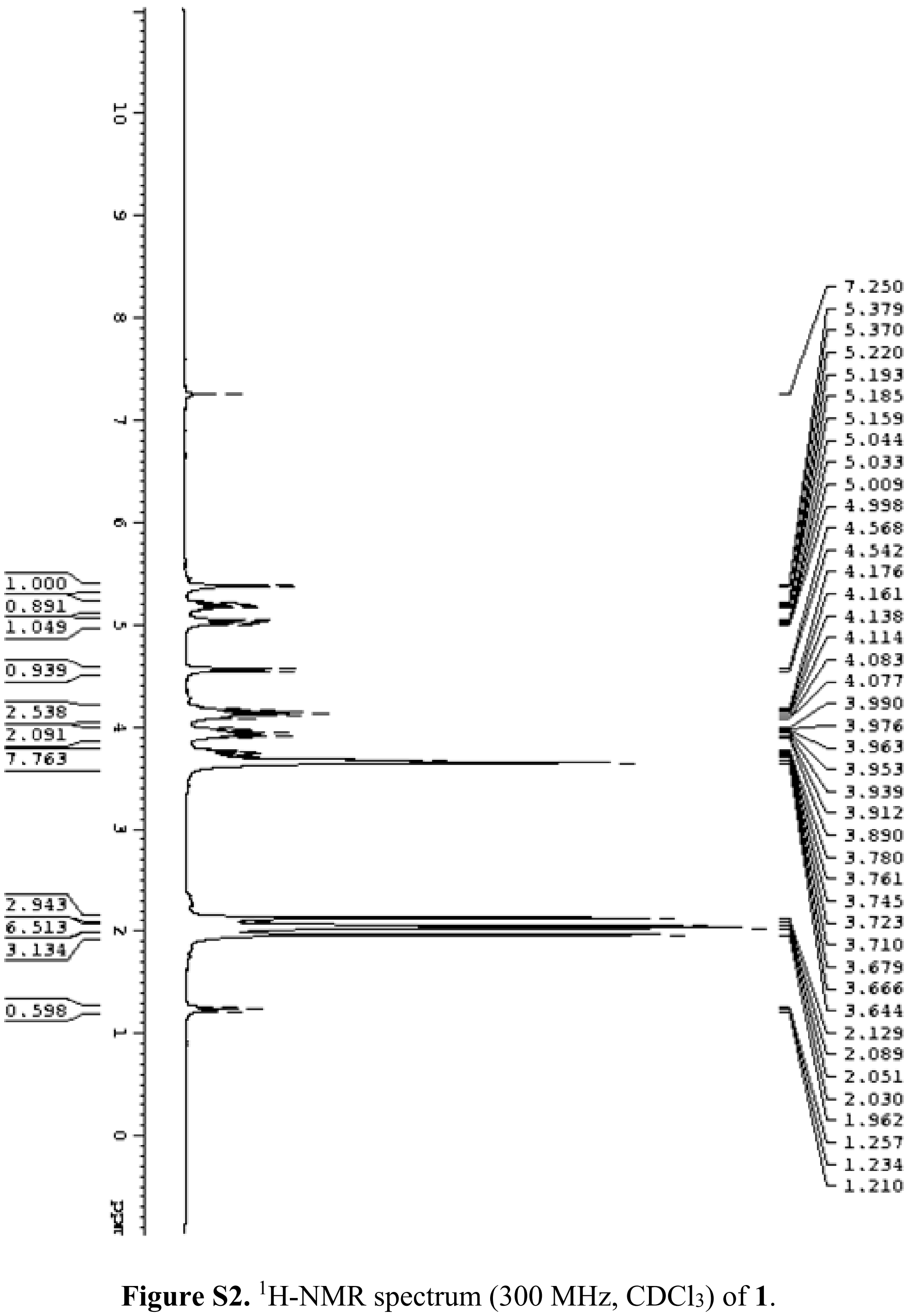

Figure S2. ^{1}H-NMR spectrum (300 MHz, $CDCl_3$) of **1**.

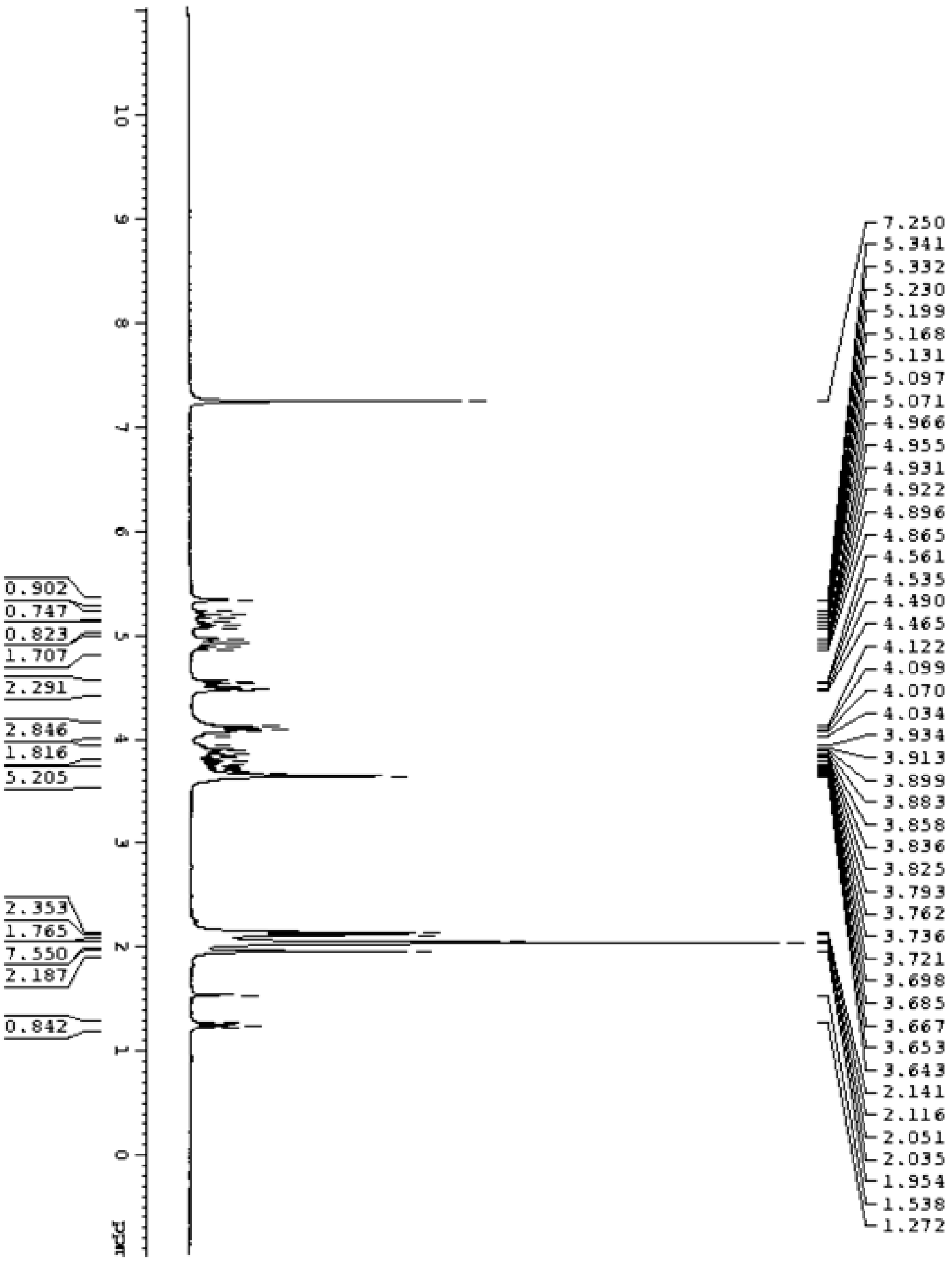

Figure S3. ^{1}H-NMR spectrum (300 MHz, $CDCl_3$) of **3**.

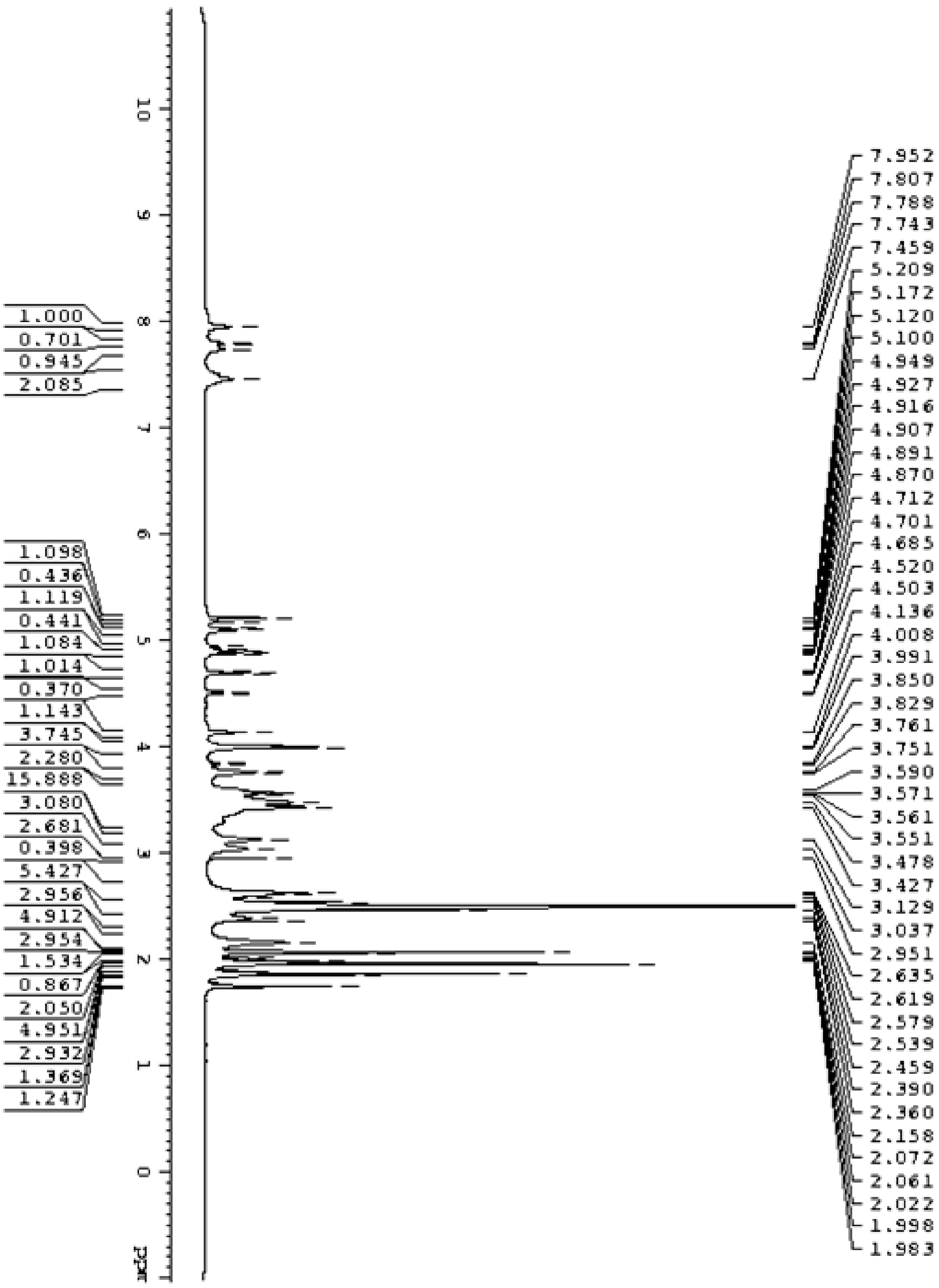

Figure S4. ^{1}H-NMR spectrum (500 MHz, d_6-DMSO) of **7b**. (acetylated).

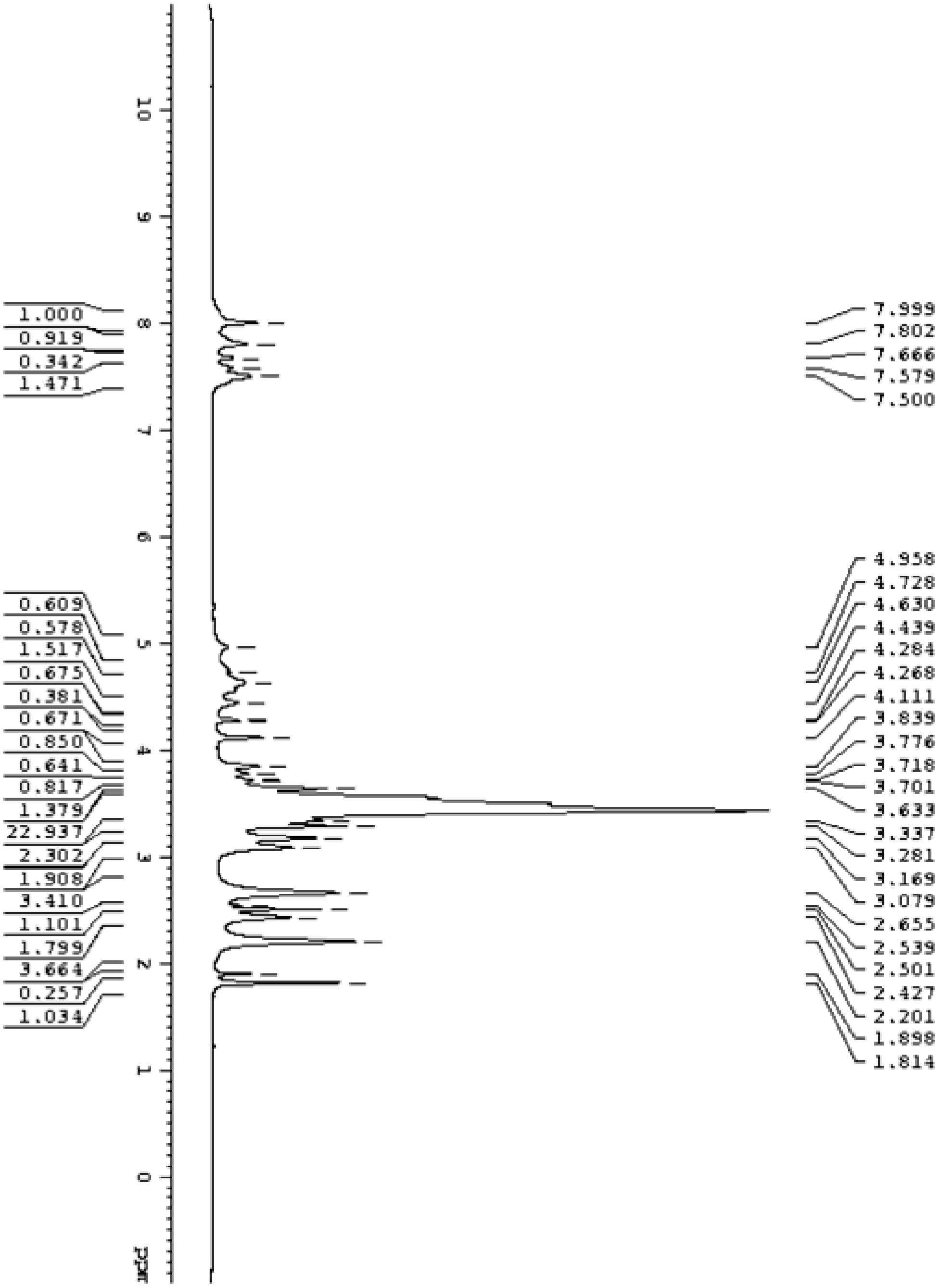

Figure S5. ^{1}H-NMR spectrum (500 MHz, d_6-DMSO) of **7b**. (deacetylated).

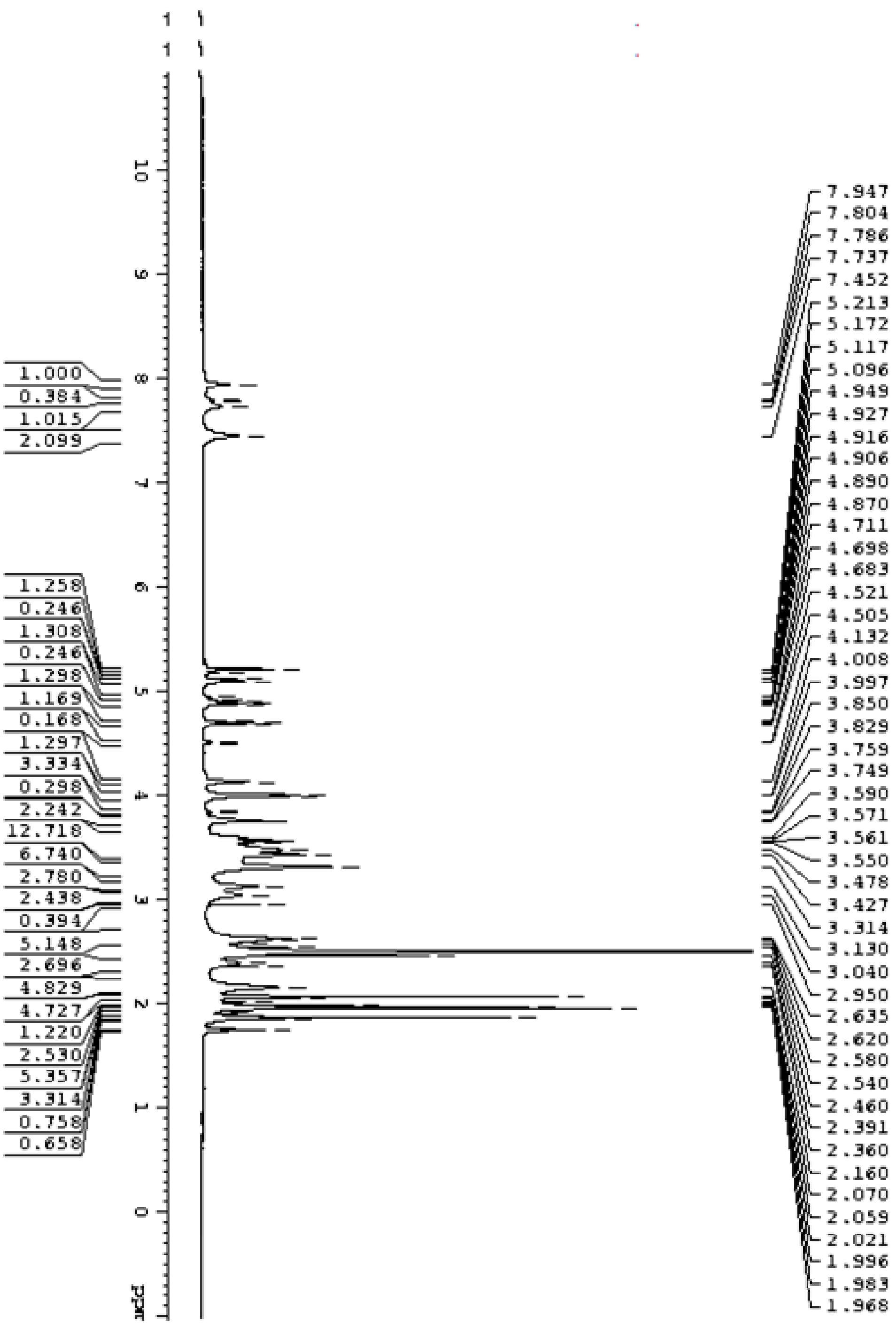

Figure S6. ^{1}H-NMR spectrum (500 MHz, d_6-DMSO) of **8a** (acetylated).

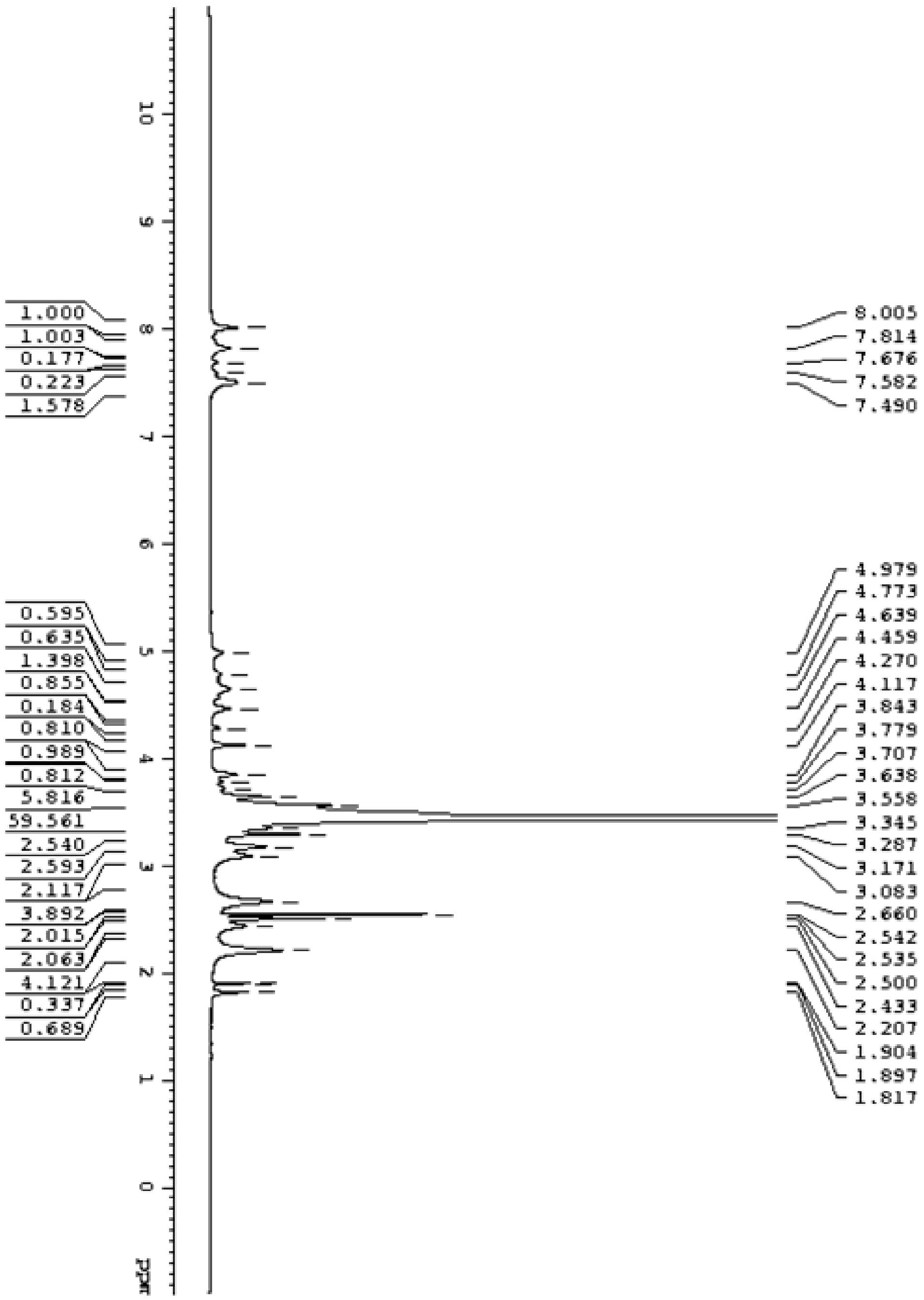

Figure S7. ^{1}H-NMR spectrum (500 MHz, d_6-DMSO) of **8a**. (deacetylated).

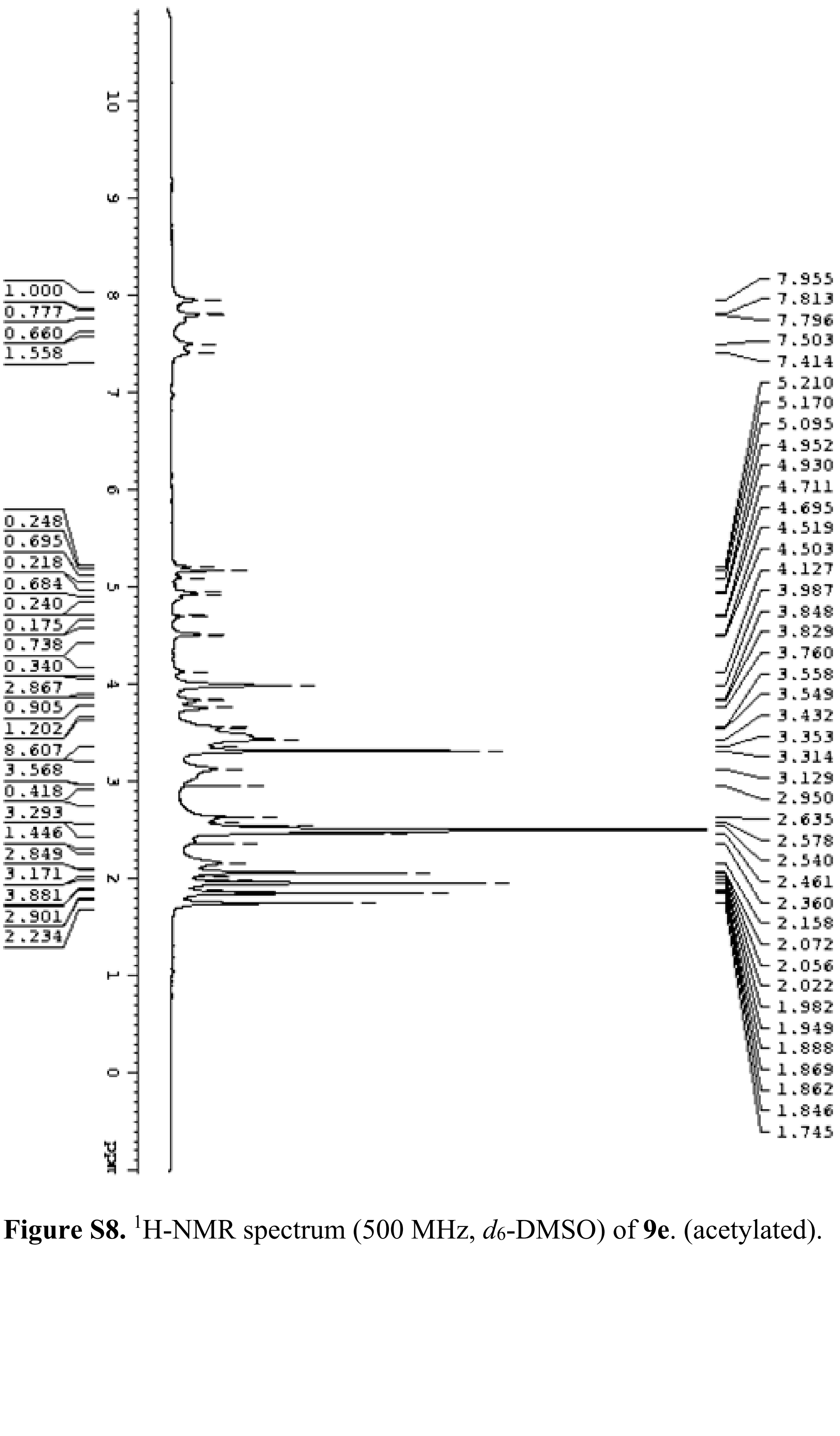

Figure S8. ^{1}H-NMR spectrum (500 MHz, *d*$_6$-DMSO) of **9e**. (acetylated).

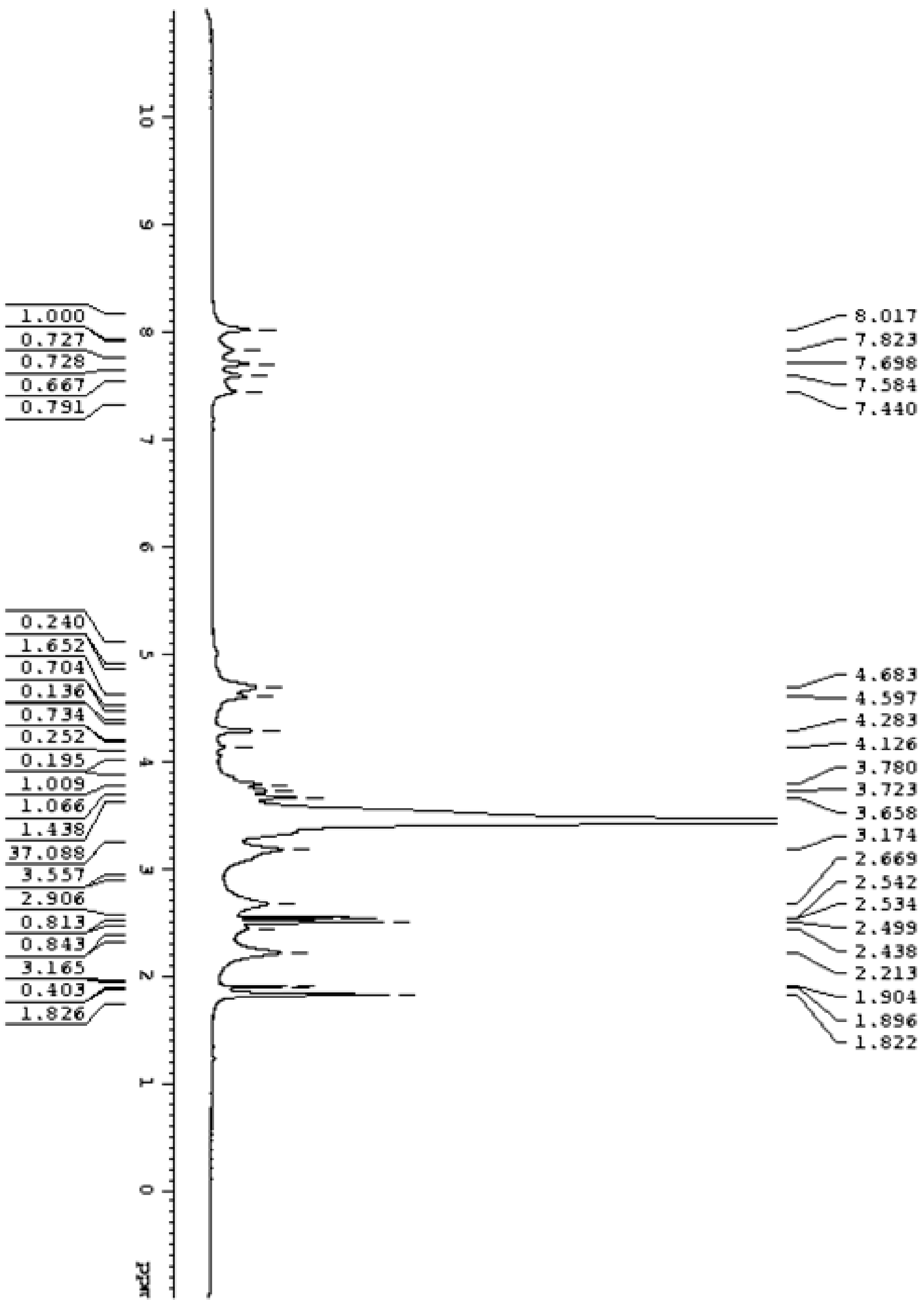

Figure S9. ^{1}H-NMR spectrum (500 MHz, d_6-DMSO) of **9e**. (deacetylated).

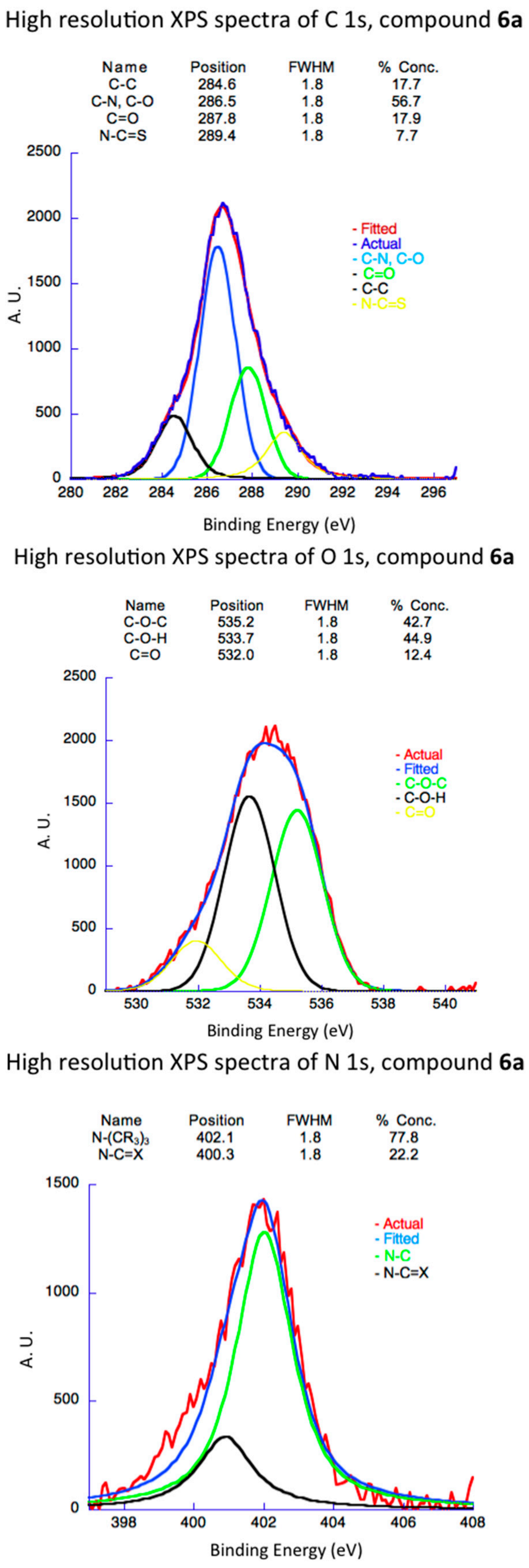

Figure S10. High resolution XPS spectra for compound **6a**.

Low resolution XPS spectrum Compound **4a**

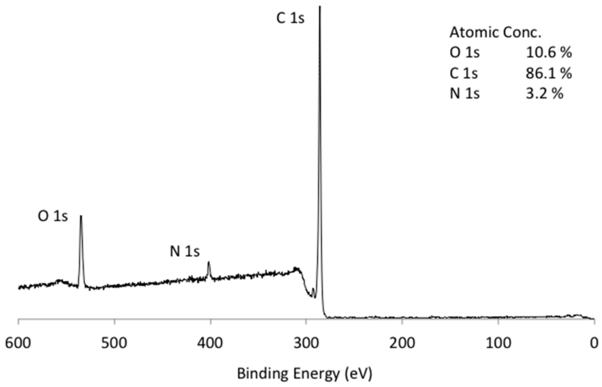

Low resolution XPS spectrum Compound **5a**

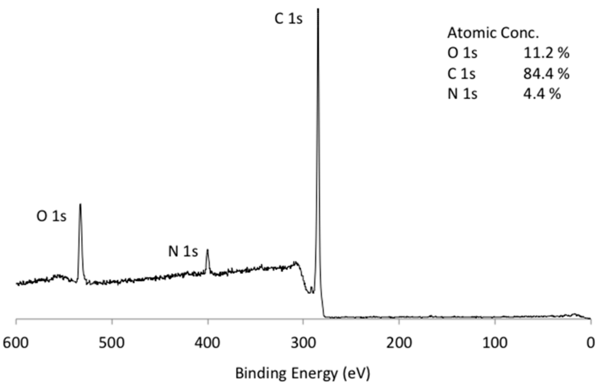

Low resolution XPS spectrum Compound **6a**

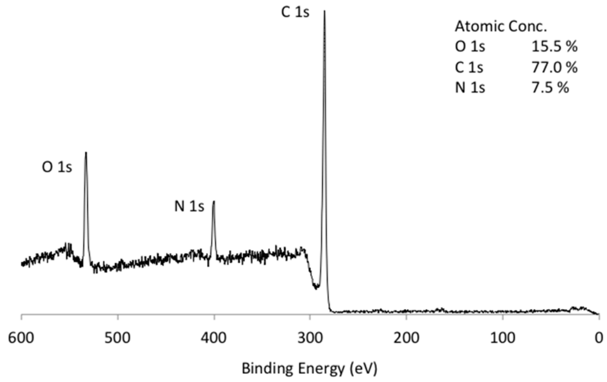

Low resolution XPS spectrum Compound **7a**

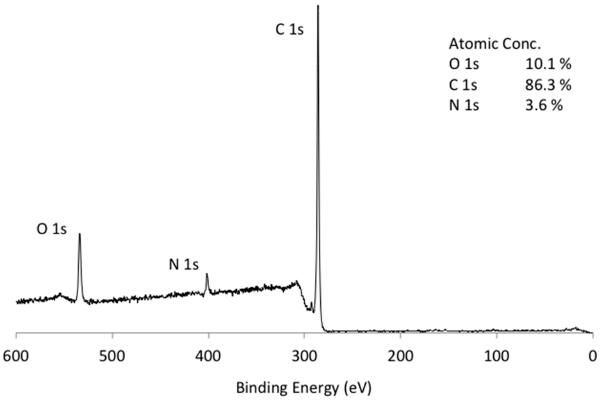

Low resolution XPS spectrum Compound **8a**

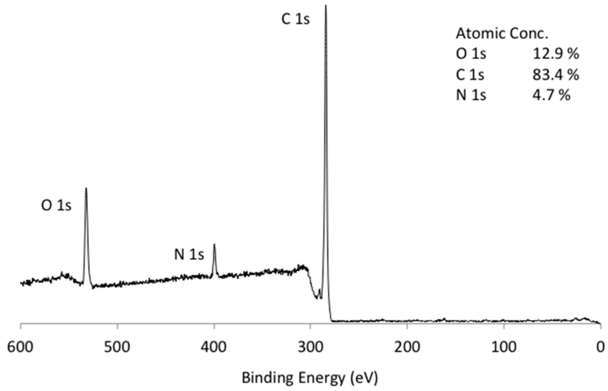

Low resolution XPS spectrum Compound **9a**

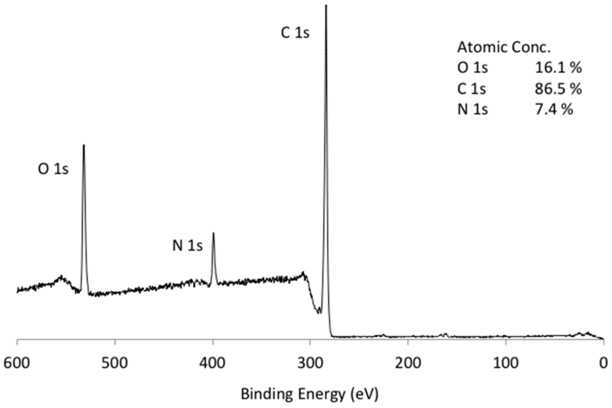

Figure S11. XPS Spectra, series **a**.

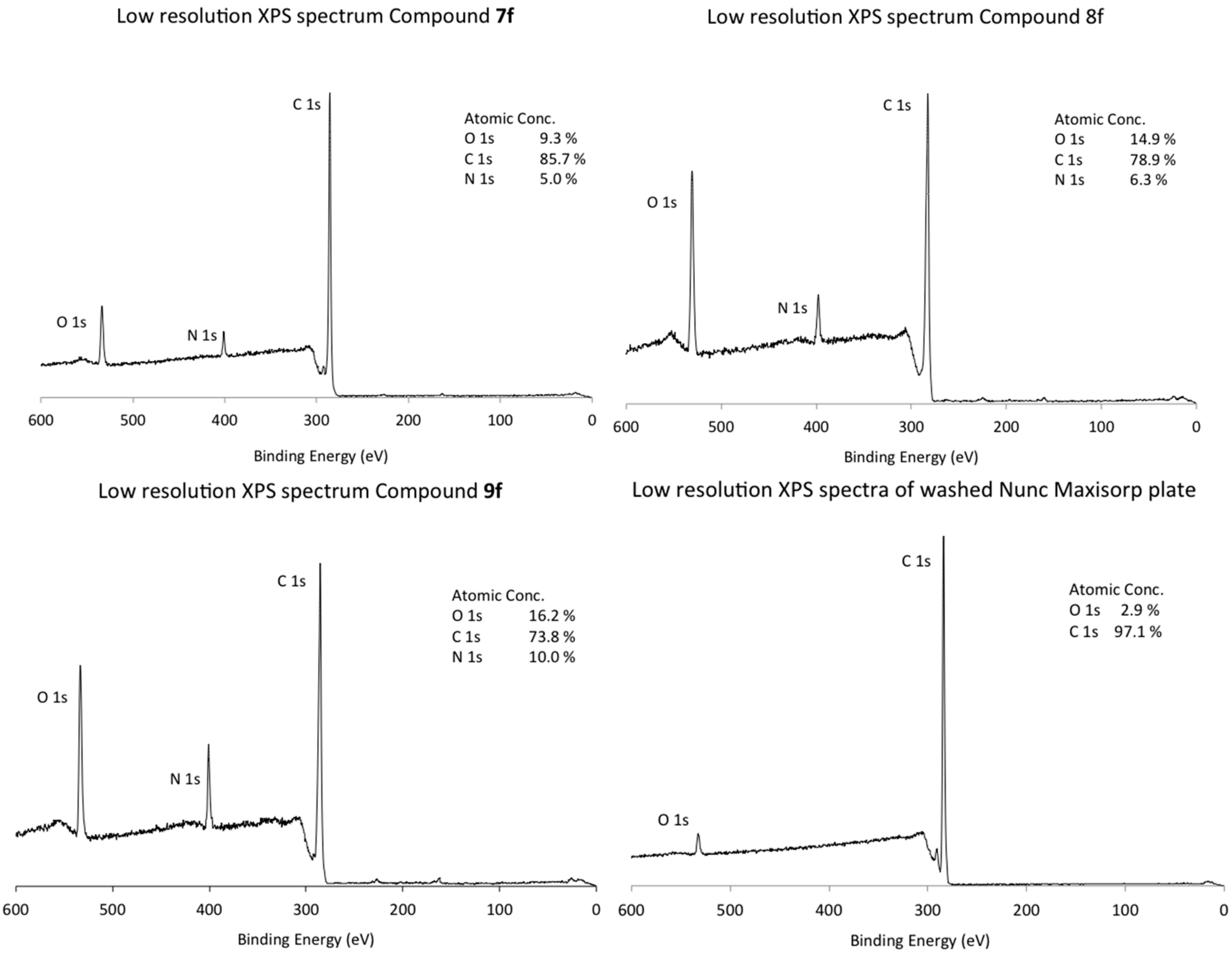

Figure S12. XPS Spectra, series **f** and control.
